# Extracellular vesicles of the probiotic bacteria *E. coli* O83 activate innate immunity and prevent allergy in mice

**DOI:** 10.1186/s12964-023-01329-4

**Published:** 2023-10-20

**Authors:** Anna Marlene Schmid, Agnieszka Razim, Magdalena Wysmołek, Daniela Kerekes, Melissa Haunstetter, Paul Kohl, Georgii Brazhnikov, Nora Geissler, Michael Thaler, Eliška Krčmářová, Martin Šindelář, Tamara Weinmayer, Jiří Hrdý, Katy Schmidt, Peter Nejsum, Bradley Whitehead, Johan Palmfeldt, Stefan Schild, Aleksandra Inić-Kanada, Ursula Wiedermann, Irma Schabussova

**Affiliations:** 1 Institute of Specific Prophylaxis and Tropical Medicine, Center for Pathophysiology, Infectiology and Immunology, Medical University of Vienna, 1090 Vienna, Austria; 2 Hirszfeld Institute of Immunology and Experimental Therapy, Polish Academy of Sciences, Wroclaw, Poland; 3 Institute of Molecular Biosciences, Karl-Franzens-University, Graz, Austria; 4 Institute of Immunology and Microbiology, First Faculty of Medicine, Charles University, and General University Hospital, Prague, Czech Republic; 5 Department of Experimental Biology, Faculty of Science, Masaryk University, Brno, Czech Republic; 6 Core Facility for Cell Imaging and Ultrastructural Research, Faculty of Life Sciences, University of Vienna, Vienna, Austria; 7 Department of Infectious Diseases, Aarhus University Hospital, Aarhus, Denmark; 8 Department of Clinical Medicine, Aarhus University, Aarhus, Denmark; 9 BioTechMed, Graz, Austria; 10 Field of Excellence Biohealth – University of Graz, Graz, Austria

**Keywords:** Allergic airway inflammation, Extracellular vesicles, Outer membrane vesicles, Microbiota and innate immunity, Nasal route of administration

## Abstract

**Background:**

*E. coli* O83 (Colinfant Newborn) is a Gram-negative (G-) probiotic bacterium used in the clinic. When administered orally, it reduces allergic sensitisation but not allergic asthma. Intranasal administration offers a non-invasive and convenient delivery method. This route bypasses the gastrointestinal tract and provides direct access to the airways, which are the target of asthma prevention. G- bacteria such as *E. coli* O83 release outer membrane vesicles (OMVs) to communicate with the environment. Here we investigate whether intranasally administered *E. coli* O83 OMVs (EcO83-OMVs) can reduce allergic airway inflammation in mice.

**Methods:**

EcO83-OMVs were isolated by ultracentrifugation and characterised their number, morphology (shape and size), composition (proteins and lipopolysaccharide; LPS), recognition by innate receptors (using transfected HEK293 cells) and immunomodulatory potential (in naïve splenocytes and bone marrow-derived dendritic cells; BMDCs). Their allergy-preventive effect was investigated in a mouse model of ovalbumin-induced allergic airway inflammation.

**Results:**

EcO83-OMVs are spherical nanoparticles with a size of about 110 nm. They contain LPS and protein cargo. We identified a total of 1120 proteins, 136 of which were enriched in OMVs compared to parent bacteria. Proteins from the flagellum dominated. OMVs activated the pattern recognition receptors TLR2/4/5 as well as NOD1 and NOD2. EcO83-OMVs induced the production of pro- and anti-inflammatory cytokines in splenocytes and BMDCs. Intranasal administration of EcO83-OMVs inhibited airway hyperresponsiveness, and decreased airway eosinophilia, Th2 cytokine production and mucus secretion.

**Conclusions:**

We demonstrate for the first time that intranasally administered OMVs from probiotic G- bacteria have an anti-allergic effect. Our study highlights the advantages of OMVs as a safe platform for the prophylactic treatment of allergy.

**Graphical Abstract:**

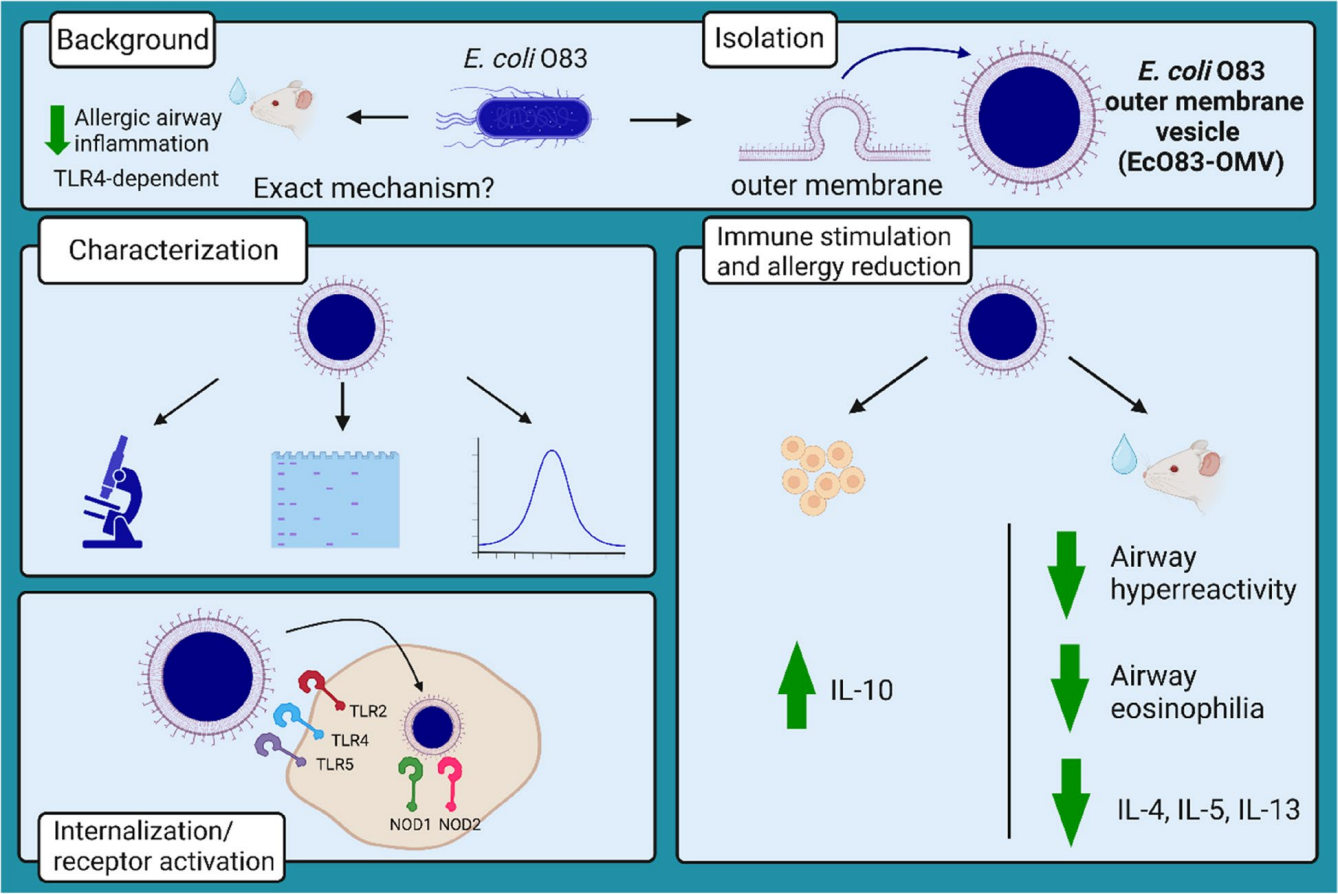

Video Abstract

**Supplementary Information:**

The online version contains supplementary material available at 10.1186/s12964-023-01329-4.

## Introduction

Allergies are one of the most common chronic inflammatory diseases, the prevalence of which has increased in industrialised countries in recent decades, resulting in a significant socioeconomic health burden [1]. To date, the only disease-modifying treatment available is allergen-specific immunotherapy. Although highly effective, it has several disadvantages such as regular administration over a long period of time and the occurrence of adverse reactions [2].

The exact mechanisms responsible for the development of allergies are largely unknown, but both genetic and environmental factors play a role [3]. The increasing prevalence of allergic diseases has been linked to changes in the microbial load in the environment due to profound changes in lifestyle and hygiene [4, 5]. Therefore, restoring a balanced microbiota at mucosal surfaces, such as the lung and gut, with probiotics is a promising strategy to prevent or treat allergies. Several clinical trials have tested the effect of oral administration of live probiotic bacterial strains in the treatment of allergic diseases [6]. Although some beneficial effects of probiotic bacteria in reducing allergic sensitisation have been reported [7, 8], the efficacy in preventing or treating allergic asthma appears inconsistent [9–11].

Studies in mice have shown that intranasal administration of *Lacticaseibacillus paracasei* NCC246 during aerosol challenge reduced the recruitment of inflammatory cells and the concentrations of IL-5 in the lungs of ovalbumin (OVA)-sensitised and challenged mice [12]. Along these lines, intranasal administration of *Lacticaseibacillus rhamnosus* GG, but not the *L. rhamnosus* strain GR-1, prevented allergic asthma in a mouse model [13]. We have shown that *Bifidobacterium longum* NCC 3001, but not *L. paracasei*, administered intranasally prior to sensitisation and challenge, suppressed allergen-specific immune responses in mice [14].

The Gram-negative (G-) probiotic bacterial strain *E. coli* A0 34/86 (*E. coli* O83; serotype O83:K24:H31) is a commercially available live oral vaccine (Colinfant Newborn). Although oral administration of *E. coli* O83 to high-risk infants reduced allergic sensitisation (skin disease), this bacterium did not provide protection against the development of respiratory allergies [15, 16]. We recently demonstrated that the route of delivery of probiotics might be important since *E. coli* O83 administered intranasally reduced allergic lung inflammation in mice. Mechanistically, the TLR4 signalling pathway was required for this beneficial effect [17].

Probiotic bacteria are generally well tolerated and considered safe. However, probiotic therapy in clinics may be associated with some risks in vulnerable target groups such as pregnant women, young children, and immunocompromised individuals [18]. Although rare, intentional supplementation of probiotics was linked with side effects such as systemic infections, toxic metabolic activities, or gene transfer [19]. Therefore, the discovery of non-living bacterial products that modulate immune function and can be conveniently used in daily life will open up new possibilities for the prevention or treatment of allergies.

G- bacteria produce extracellular vesicles called outer membrane vesicles (OMVs). They are produced during normal bacterial growth by detachment of the outer membrane and consist largely of outer membrane components such as lipopolysaccharide (LPS), lipids and proteins, but may also contain material from the periplasmic space or from within the bacterial cell, such as nucleic acids [20]. Several studies have investigated OMVs of pathogenic [21–24] or non-pathogenic [25–27] *E. coli* strains, but OMVs of *E. coli* O83 have not yet been investigated. OMVs contain microbe-associated molecular patterns that give them immunostimulatory properties. They can interact with different pattern recognition receptors in and on host cells to stimulate innate and adaptive immune responses [28]. As natural and non-replicative particles, OMVs are ideal candidates for use as safe vaccine platforms due to their nanosize (20–200 nm), low toxicity, cargo protection, ability to cross biological barriers, and the simplicity of their manufacturing and purification processes [29–31]. Although some OMVs-based vaccines against infectious diseases are already on the market [32], the potential of OMVs to treat non-communicable diseases, such as allergies, has not yet been satisfactorily investigated.

In this study, we hypothesised that non-proliferating *E. coli* O83-derived vesicles have immunomodulatory potential and can recapitulate the allergy-preventive effect of their parent strain in a mouse model of experimental asthma, providing a mucosal vaccine candidate with fewer risks associated with their use in humans.

## Materials and methods

### Bacteria

*Escherichia coli* strain A0 34/86 (*E. coli* O83; serotype O83:K24:H31) was grown in 10 ml Luria–Bertani (LB) medium (Sigma Aldrich, St. Louis, MO) as described before [17]. The culture was inoculated with a sample from a frozen glycerol stock and incubated for 3 h at 37 °C and 200 rpm. The colony-forming units (CFU) were calculated using a growth curve established in house. For experiments in cell culture, the bacteria were inactivated with 1% paraformaldehyde (PFA; SAV Liquid production, Flintsbach, Germany) for 3 h at room temperature (RT), washed twice in phosphate-buffered saline (PBS; Gibco, Thermo Scientific, Waltham, MA) and stored at -20 °C until further use. For the preparation of the lysate, 10 ml overnight culture of *E. coli* O83 in brain heart infusion broth (BHI; Sigma Aldrich, St. Louis, MO) was centrifuged (10 min at 4 °C and 3 000 × g) and the pellet was washed twice with PBS. The washed pellet was then dissolved in 1 ml of lysis buffer (20 mM Tris–HCl, 0.14 M NaCl, 10% glycerol; pH = 7) containing protease inhibitor cocktail (Roche, Mannheim, Germany) and then disrupted in a Precellys tissue homogenizer using microorganism disruption beads (both from Bertin Technologies, Montigny-le-Bretonneux, France) (30 s at RT and 6 500 rpm). The supernatant was kept at -20 °C until further use.

### Isolation of *E. coli* O83-derived outer membrane vesicles (EcO83-OMVs)

Vesicles were isolated according to published protocol [33]. Briefly, *E. coli* O83 was grown in BHI in an overnight culture at 37 °C and 180 rpm. The main culture was inoculated 1:100 from the overnight culture and grown for 8 h at the same conditions. The culture was centrifuged for 15 min at 4 °C and 10 000 × g. The supernatant was filtered through a 0.22 µm-pore-size filter (Merck Millipore, Burlington, MA). OMVs were isolated by ultracentrifugation (4 h at 4 °C and 150 000 × g; Beckman Coulter 45Ti rotor). The pellet was dissolved in 500 µl of sterile 0.9% NaCl (B. Braun, Maria Enzersdorf, Austria). OMVs were stored at 4 °C for immediate use or at -20 °C for long-term storage.

### Protein and endotoxin analysis

Protein concentration was determined by Bradford protein assay (BioRad, Hercules, CA) and the endotoxin content was analysed using the PyroGene™ Recombinant Factor C Endpoint Fluorescent Assay (Lonza, Basel, Switzerland) according to manufacturer’s instructions. The absorbance and fluorescence were measured with TECAN Spark 10 M plate reader (Tecan Trading AG, Switzerland). The protein profile of OMV samples was analysed by Agilent Bioanalyzer 2100 with the Protein 230 kit (Agilent Technologies, CA, USA) according to manufacturer’s instructions.

### Transmission electron microscopy (TEM)

The bacterial culture sample taken before filtration and centrifugation and the isolated EcO83-OMVs were imaged with TEM. The bacterial culture was centrifuged at 5 000 × g and the pellet was washed twice with PBS before microscopy. EcO83-OMVs were dissolved in NaCl. Ultrastructural visualization was carried out by single-droplet negative stain. Briefly, 5 µl of each OMV preparation were left to adhere on formvar- and carbon-coated grids for 20 s. Simultaneous addition of 5—10 µl 1% uranylacetate and absorption of liquid was repeated twice to contrast the samples. Dried grids were imaged in an FEI Tecnai20 electron microscope (FEI, Eindhoven, NL) equipped with a 4 K Eagle-CCD camera.

### Liquid chromatography/mass spectrometry (LC–MS) analyses of proteins

The label-free proteomics analyses were performed as previously described [34]. Briefly, 20 μg of protein was dissolved in SDS-PAGE loading buffer, subjected to SDS-PAGE followed by reduction, blocking of reduced cysteine residues and in-gel trypsin digestion. Resulting peptides were purified with PepClean™ C18 Spin Columns and dried on a miVac Duo Concentrator (Genevac, Ipswich, UK). Samples were stored at -20 °C until LC–MS/MS analyses. Peptide samples were analysed by nano LC (Easy-nLC 1200, Thermo Scientific, Waltham, MA)-tandem MS (Q-Exactive HF-X Hybrid Quadrupole Orbitrap, Thermo Scientific, Waltham, MA). Proteins were identified and quantified using MaxQuant [35] (version 1.5.3.30) with its Andromeda algorithm against the *E. coli* (strain K12) sequence database (*E. coli* proteome with 4448 reviewed sequences downloaded from UniProt database, https://www.uniprot.org/). The “match between runs” was applied and LFQ data were used for quantification using Perseus v1.6 [36]. Values below detection limit were assumed to be zero. A fold change of two or higher was considered for enrichment. The Venn diagram was created using DeepVenn (arXiv:2210.04597) and the volcano plot was created using VolcaNoseR [37]. The MS proteomics data have been deposited to the ProteomeXchange Consortium via the PRIDE partner repository [38] with the dataset identifier PXD040963.

### Culture and stimulation of human embryonic kidney (HEK) 293 cells

Recognition of EcO83-OMVs by innate pattern recognition receptors was tested on HEK 293 cells transfected with human receptors hNOD1, hNOD2, hTLR2, hTLR5 and hTLR4/CD14/MD2. Cells were cultured according to published protocols [39] using the appropriate culture media (Table S1). Briefly, cells were stored in liquid nitrogen in freezing medium containing 10% DMSO (Merck, Darmstadt, Germany). The cells were thawed and transferred to the required growth medium (Table S1). Cells were grown at 37 °C and 5% CO_2_. Generally, the growth medium was changed every 2–3 days and cultures were split when they reached ~ 80% confluence. HEK-293/hTLR4/CD14/MD2 cells were grown in cell culture flasks for challenging adherent cells (Sarstedt, Nürnbrecht, Germany). HEK-293 cells were seeded at the density of 4 × 10^5^ cells/ml, 100 µl/well, in sterile 96-well flat bottom plates and incubated at 37 °C in the respective growth media (Table S1) overnight before the stimulation. The cells were stimulated with EcO83-OMVs (1 ng/ml, 10 ng/ml, 100 ng/m, and 1000 ng/ml), PFA-fixed *E. coli* O83 (10^7^ CFU/ml) and the respective positive control Pam3CysSerLys4 (Pam3CSK4; 1 µg/ml) for TLR2, ultrapure LPS (1 µg/ml) for TLR4, recombinant flagellin protein from *Salmonella typhimurium* (recFLA-ST; 100 ng/ml) for TLR5, L-Ala-γ-D-Glu-mDAP (Tri-DAP; 5 µg/ml) for NOD1 and muramyl dipeptide (MDP; 5 µg/ml) for NOD2 (all from Invivogen, Toulouse, France) at 37 °C and 5% CO_2_ for 24 h. Untreated cells were used as negative controls. The activation of a specific receptor was confirmed by measuring IL-8 in the cell culture supernatants.

### Mice

Wild type BALB/c mice (female, aged 6–8 weeks) were purchased from Charles River (Sulzfeld, Germany). Animals were kept in conventional housing at the Institute for Pathophysiology and Allergy Research of the Medical University of Vienna with free access to food and water. Experiments were approved by the Animal Experimentation Committee of the Medical University of Vienna and the Austrian Federal Ministry of Education, Science and Culture (BMBWF-66.009/0277-V/3b/2019).

### Isolation and stimulation of splenocytes and bone marrow-derived dendritic cells (BMDCs) derived from naïve mice

Spleens from naïve mice were isolated, cultured, stimulated and supernatants were analysed as previously described [40]. Briefly, they were collected in RPMI 1640 media containing 100 µg/ml gentamycin, pushed through a metal net and the cell suspension was then pipetted through a 70 µm cell strainer. Samples were incubated in ACK Lysing Buffer (Lonza, Basel, Switzerland) for 1.5 min to lyse erythrocytes and cells were resuspended in complete RPMI medium (Table S1). 5 × 10^5^ cells/well were plated in 96-well plates and stimulated with EcO83-OMVs (1 ng/ml, 10 ng/ml, and 100 ng/ml), PFA fixed *E. coli* O83 (10^6^, 10^7^, and 10^8^ CFU /ml) and LPS (1 µg/ml;) at 37 °C and 5% CO_2_ for 72 h. Cell culture supernatants were collected and cytokines were analysed. The isolation and differentiation of the bone marrow cells into BMDCs was performed as described previously [41]. In short, femur and tibia were extracted from BALB/c mice. The bones were cleaned from any remaining tissue and washed in 70% ethanol by shaking for one min followed by rinsing twice in PBS. Both ends of the bones were cut off and the bone marrow was flushed out with 2.5 mL BMDC medium (Table S1) from each side. The cell suspension was then pipetted through a 70 µm cell strainer, red blood cells were lysed using ACK lysing buffer and cells were re-suspended in BMDC medium containing 20 ng/mL recombinant granulocyte macrophage colony-stimulating factor (rGM-CSF; Peprotech, London, UK). Cells were seeded in a Petri dish (2 × 10^5^ cells/ dish in 10 ml medium) and maintained as follows: on day 3, 10 ml of DC-medium with rGM-CSF were added to each dish; on day 6, 10 ml were taken out of each dish and replaced by fresh DC-medium with rGM-CSF; on day 9, cells were harvested by centrifugation, and seeded at 1 × 10^7^ cells/ml in 48-well flat bottom plates. The cells were stimulated for 24 h with the stimuli indicated above for the splenocytes and the cytokines were measured in the cell culture supernatants.

### Allergic sensitisation, allergic challenge, and intranasal treatment with EcO83-OMVs

Airway inflammation was induced in female 6–8 weeks old BALB/c mice with ovalbumin (OVA; grade V; Sigma-Aldrich, St. Louis, MO) as previously described [17]. Briefly, mice were intraperitoneally (i.p) sensitized with 10 µg OVA and 65 µg aluminiumhydroxide (alum; SERVA Electrophoresis, Heidelberg, Germany) or PBS/alum in 150 µl on days 0 and 14. On days 21–24, mice were anesthetized with 5% isoflurane (CP-pharma, Burgdorf, Germany) and treated with 100 µg OVA in 30 µl PBS intranasally (i.n.) or 30 µl PBS alone. Thirty minutes prior to each OVA application, mice received i.n. 0.1 µg (group OMVs 0.1 µg/OVA), 1 µg of EcO83-OMVs (group OMVs 1 µg/OVA), or 0.9% NaCl (groups Sham/PBS and Sham/OVA). On day 25, airway hyperresponsiveness was tested using unrestrained whole-body plethysmography (Buxco® Small Animal Whole-body Plethysmography, Data Sciences International; St Paul, MN). Mice were subjected to increasing doses (0–50 mg/mL) of nebulized methacholine (Sigma-Aldrich, St. Louis, MO) and the enhanced pause was measured as an index for airway obstruction. Mice were terminally anesthetized on day 26, spleens and lungs were harvested and bronchoalveolar lavage (BAL) and blood were taken.

### Characterisation of cell populations in the BAL

Lungs were lavaged twice with 500 µl of ice-cold PBS, and cells were collected by centrifugation. The pellet was resuspended in 200 µL PBS and 4 × 10^4^ cells were spun onto a microscopic slide (Shandon Cytospin; Shandon Southern Instruments, Waltham, MA), air-dried and stained with haematoxylin and eosin (Hemacolor; Merck, Darmstadt, Germany). Slides were scanned using the Tissue FAXSi Plus system and cells were counted in Tissue FAXS viewer (TissueGnostics, Vienna, Austria).

### Lung histology and isolation and restimulation of lung cells ex vivo

Histological evaluation of lungs and isolation, stimulation, and analysis of pulmonary single cell suspensions were performed according to published protocol [40]. Briefly, lungs were fixed in 7.5% formaldehyde (SAV Liquid production, Flintsbach, Germany) and the tissue was embedded in paraffin (Carl Roth, Karlsruhe, Germany), cut into three µm sections and stained using haematoxylin and eosin (H&E) and periodic acid-Schiff (PAS, Sigma-Aldrich). Pathology score was evaluated based on a scoring system adapted from Zaiss et al*.*[42] by analysing 5 independent regions on each sample (H&E: inflammatory cells in peribronchial sites: 0 = no inflammation; 1 = few inflammatory cells; 2 = layer of 1–4 inflammatory cells; 3 = layer of 4–10 inflammatory cells; 4 = layer of more than 10 inflammatory cells; PAS: mucus secretion and presence of goblet cells: 0 = no goblet cells; 1 = mild mucus secretion, 1/3 of epithelium occupied by goblet cells; 2 = moderate mucus secretion, 1/3–2/3 of epithelium occupied by goblet cells; 3 = high mucus secretion and > 2/3 of epithelium occupied by goblet cells).

### Isolation and restimulation of lung cells ex vivo

Lungs were minced and incubated in 6 ml RPMI 1640 (Biowest, Nuaillé, France) containing 100 µg/ml gentamycin (Carl Roth, Karlsruhe, Germany), 10 µg/mL liberase TL (Roche, Mannheim, Germany) and 0.5 mg/mL DNAse (Sigma-Aldrich, St. Louis, MO) at 37 °C and 5% CO_2_ for 45 min. The lung pieces where then pushed through a 70 µm cell strainer and red blood cells were lysed as described above. Cells were resuspended in complete RPMI (Table S1) and cells were plated at a concentration of 5 × 10^6^ cells/ml in 96-well plates. Cells were stimulated with endotoxin-free OVA (100 µg/ml; EndoGrade; Hyglos, Bernried am Starnberger See, Germany) at 37 °C and 5% CO_2_ for 72 h. Cell culture supernatants were collected and cytokines were analysed.

### ELISA

For detection of cytokines, eBioscience Ready-SET-Go! ELISA kits (eBioscience, San Diego, CA) were used according to manufacturer’s instructions. Briefly, a 96-well-plate (Nunc MaxiSorp™, Thermo Scientific, Waltham, MA) was coated with the provided capture antibody and incubated at 4 °C overnight. The plate was washed 3X with PBS containing 0.05% Tween 20 (Carl Roth, Karlsruhe, Germany) (PBST). The plate was blocked with 200 µl of the kit’s ELISA/ELISPOT diluent at RT for 1 h. Samples and standards were diluted in the same diluent, 50 µl were added to the plate and incubated at 4 °C overnight. Technical triplicates of the samples were used in all assays. This and all subsequent steps were followed by a washing step as described above. Biotinylated detection antibody was incubated at RT for 1 h. Then, horseradish-peroxidase conjugated avidin was added and incubated at RT for 30 min, and for detection, the substrate TMB was used. The reaction was stopped using 0.18 M H_2_SO_4_ (Carl Roth, Karlsruhe, Germany) and the absorbance at 450/570 nm was measured in a TECAN Spark™10 M plate reader.

### Statistics

Statistical analysis was performed in GraphPad Prism Software 9 (GraphPad Software Inc.) using One-Way ANOVA or Two-Way ANOVA followed by a post-hoc Tukey’s multiple comparison test. Data are shown as mean ± SD. Significant differences are marked as *p < 0.5; **p < 0.05; ***p < 0.01, ****p < 0.001.

## Results

### Probiotic *E. coli* O83 produces outer membrane vesicles

**Fig. 1:**
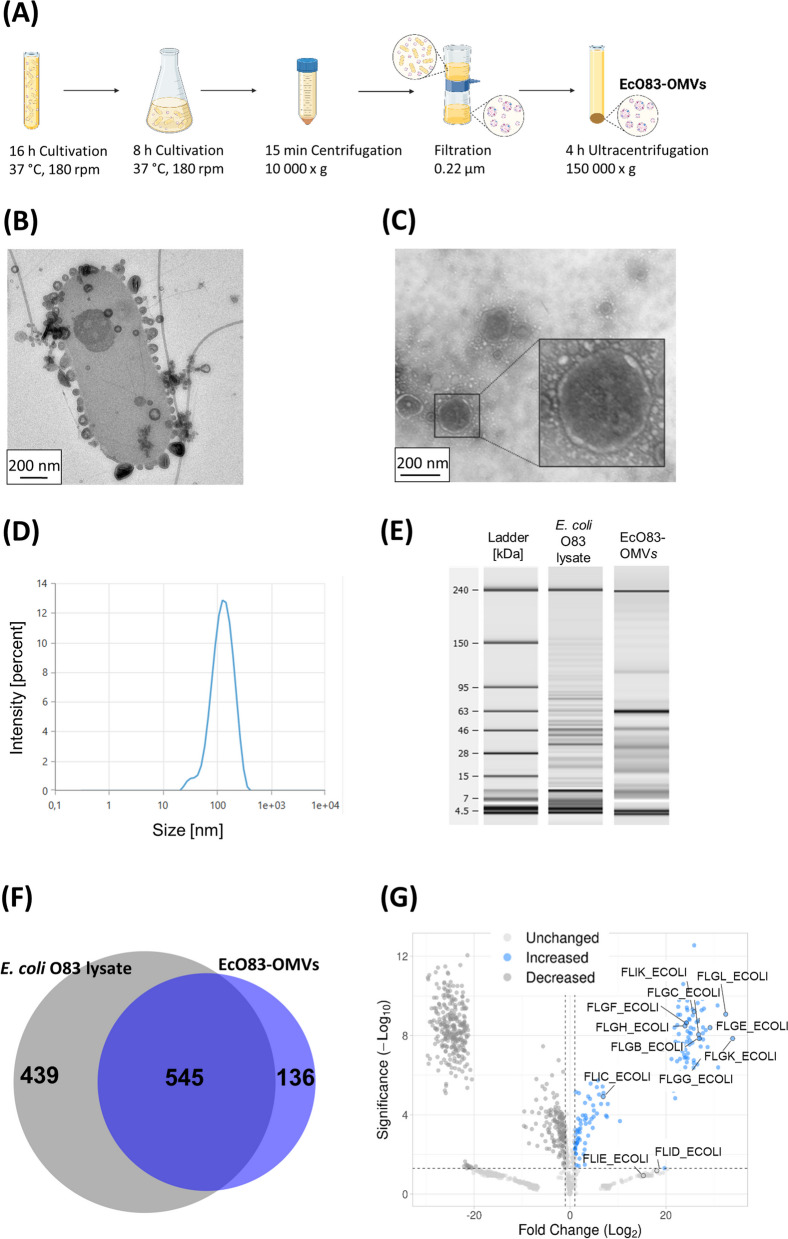
Isolation and characterisation of EcO83-OMVs. **A** OMVs were isolated from the culture supernatant of *E. coli* O83 by ultracentrifugation. **B** Visualisation of the budding process of EcO83-OMVs in the sample taken before filtration and centrifugation by TEM. **C** Visualisation of the isolated EcO83-OMVs by TEM. Scale bar = 200 nm. **D** The size of the isolated EcO83-OMVs were determined by Dynamic Light Scattering. **E** Protein profiles of *E. coli* O83 lysate and EcO83-OMVs analysed by Bioanalyzer. The unaltered original picture is shown in Fig. S1. **F** Intersection of proteins associated with *E. coli* O83 lysate and EcO83-OMVs depicted by Venn diagram. **G** Volcano plot visualization of enriched proteins in EcO83-OMVs compared to *E. coli* O83 lysate with annotated flagella proteins

EcO83-OMVs were isolated from cell-free culture supernatants by ultracentrifugation (Fig. 1A). In parallel, we processed the vesicle-free supernatant and mock control (Supplementary Material and Methods). To exclude the possibility of contamination of purified vesicles with live bacteria, EcO83-OMVs and both control samples were plated on agar plates and examined for the presence of bacterial colonies. Even after 96 h of culture, no bacterial colonies were detectable on the plates with neither of the sample (Figure S1). TEM visualization showed that EcO83-OMVs are spherical structures with a diameter of approximately 100 to 200 nm (Fig. 1B and C). Figure 1B shows a TEM image of budding vesicles on the surface of the parent bacteria and their release into the surrounding medium. This sample was taken before the purification process. Dynamic light scattering (DLS) measurement revealed a high concentration of vesicles with a mean size around 110 nm (Fig. 1D). In total, we isolated 7 × 10^12^ particles from a two litre culture of *E. coli* O83 (O.D. = 2) (Table S2). To compare the protein profile of the vesicles with that of the parental bacteria, we performed automated electrophoresis with the Bioanalyzer. The lysate of the whole bacteria and the OMVs had different protein profiles (Fig. 1E and S2). Several prominent bands of about 19, 34, 49 and 64 kDa, can be identified in the OMV sample (Fig. 1E; Table S3). The most dominant protein had a size of 64 kDa and accounted for 27.5% of the total protein content (Table S3). We performed a LC–MS-based label-free protein quantification to compare the protein expression profile in *E. coli* O83 bacteria and EcO83-OMVs in more detail. While most of the identified proteins (545) are shared by the OMVs and the parent bacteria, there is a group of 136 proteins (Fig. 1F), including flagellar proteins FlgL 1 and 3, FlgE, FlgG, and FliC, detected exclusively in the vesicles (Fig. 1G and Table S4). The molecular mass of flagellin ranges from 37 to 67 kDa depending on the *E. coli* strain [43]. Our preliminary sequencing data confirm that the band of 64 kDa is *E. coli* O83 flagellin (data not shown). LPS is the major microbe-associated molecular pattern of the outer membrane of Gram- bacteria and we have shown that LPS content in OMVs is approximately 5 times higher than the protein content (Table S2).

### EcO83-OMVs activate the surface receptors TLR4, TLR2, and TLR5 and the intracellular receptors NOD1 and NOD2

**Fig. 2:**
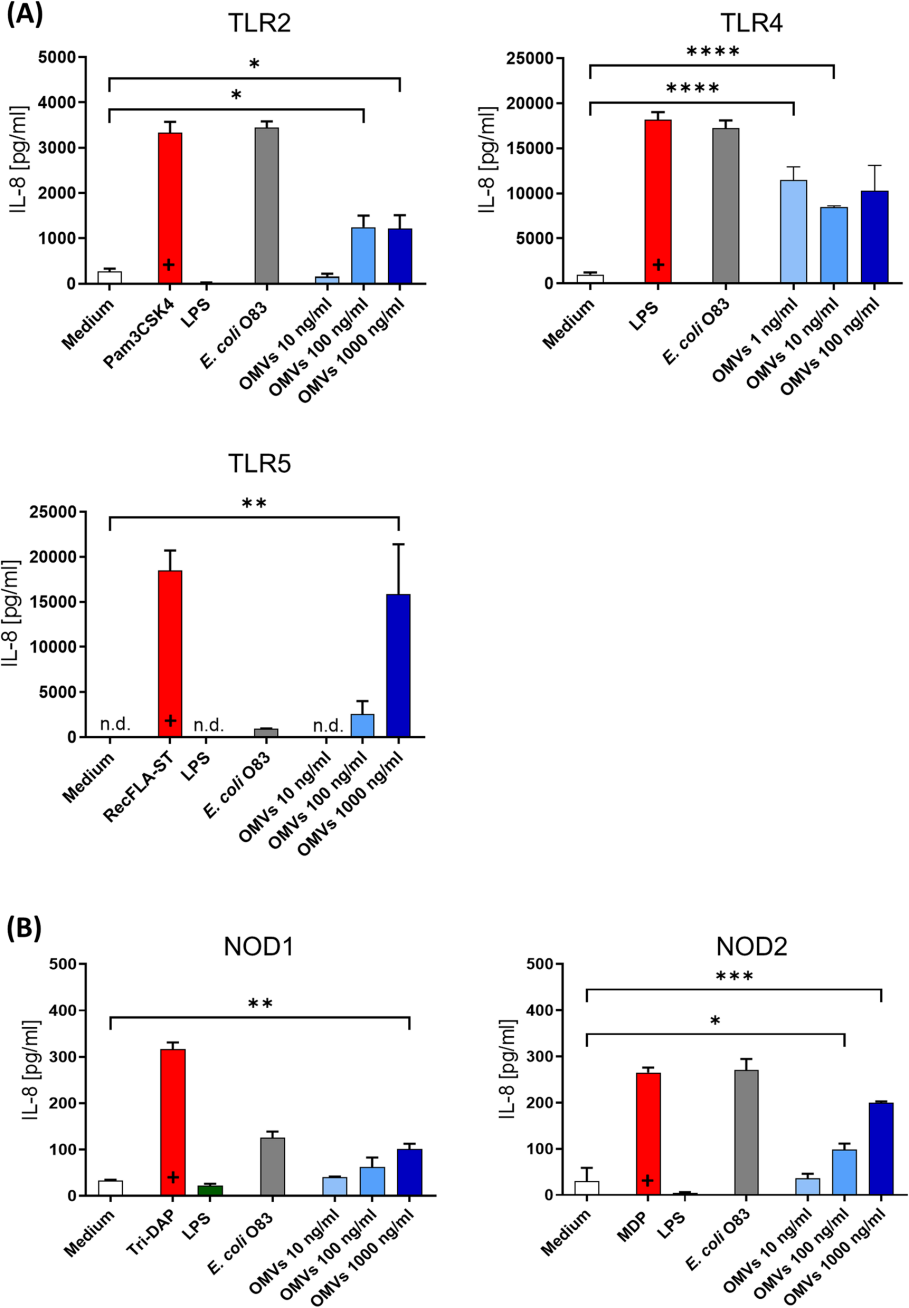
Receptor activation by EcO83-OMVs. HEK-293 cells expressing (**A**) hTLR2, hTLR4 and hTLR5, **B** hNOD1 and hNOD2 were treated with the respective positive control (Pam3CSK 1 µg/ml, LPS 1 µg/ml, recFLA-ST 100 ng/ml, Tri-DAP 5 µg/ml, MDP 5 µg/ml), fixed *E. coli* O83 (10^7^ CFU/ml), EcO83-OMVs (OMVs; 1 ng/ml, 10 ng/ml, and 100 ng/ml for TLR4; and 10 ng/ml, 100 ng/ml and 1000 ng/ml for TLR2, TLR5, NOD1 and NOD2) or medium only. Receptor activation was detected as an increase in IL-8 production. Data were analysed using a One-Way ANOVA followed by post-hoc Tukey’s multiple comparison test. *p < 0.5; **p < 0.05; ***p < 0.01; ****p < 0.001; +  = significant difference between positive and negative control. Data are representative of three independent experiments. Mean ± SD is shown. Significant differences between EcO83-OMVs and the negative controls are indicated n.d. = not detected

OMVs derived from G- bacteria have previously been shown to interact with the host via their cargoes such as LPS, lipoproteins and flagellin [23, 44, 45] leading to modulation of the innate immune response. We have previously shown that *E. coli* O83 prevents the development of allergies in mice and that this effect depends on its interaction with TLR4 [17]. Here we show that EcO83-OMVs, similar to the parent bacteria, target TLR4, confirming the presence of the TLR4 ligands on the bacteria and also on their vesicles (Fig. 2A). Similarly, we observed the involvement of TLR2 (lipoprotein receptor) and TLR5 (flagellin receptor) in the interaction with EcO83-OMVs (Fig. 2A). TLR2, but not TLR5, was activated by the parent bacteria, confirming our observation from proteomic analysis showing that flagellum-associated proteins are more abundant in OMVs than in the parent bacteria.

Unlike TLR2/4/5, which recognise microbial ligands on the cell surface, NOD1 and NOD2 sense bacterial products such as peptidoglycan in the cytosol [46, 47] and bacterial OMVs have been shown to deliver peptidoglycan into the cytoplasm [47]. NOD1 and NOD2 recognised EcO83-OMVs and whole bacteria lysate, and EcO83-OMVs interact with these receptors in a dose-dependent manner (Fig. 2B).

HEK-293 cells transfected with TLR2, TLR4, and TLR5 produce IL-8 when incubated with EcO83-OMVs, as detected by ELISA. IL-8 is a chemokine produced in response to TLR agonists in an NF-κB/AP-1-dependent manner [48, 49]. Since NF-κB is a crucial transcription factor for the induction of a plethora of inflammatory genes [50], we investigated whether EcO83-OMVs can induce the expression of other NF-κB target genes. RAW264.7 cells were treated with medium, LPS (1 µg/ml), *E. coli* O83 (10^7^ CFU/ml) and EcO83-OMVs (10 ng/ml and 100 ng/ml) for 6 h. The results show that EcO83-OMVs induced mRNA expression of IL-6, TNF-α and IL-1β in a dose-dependent manner, further confirming the involvement of the NF-kB pathway (Figure S3).

### EcO83-OMVs have an immunomodulatory effect in vitro

**Fig. 3:**
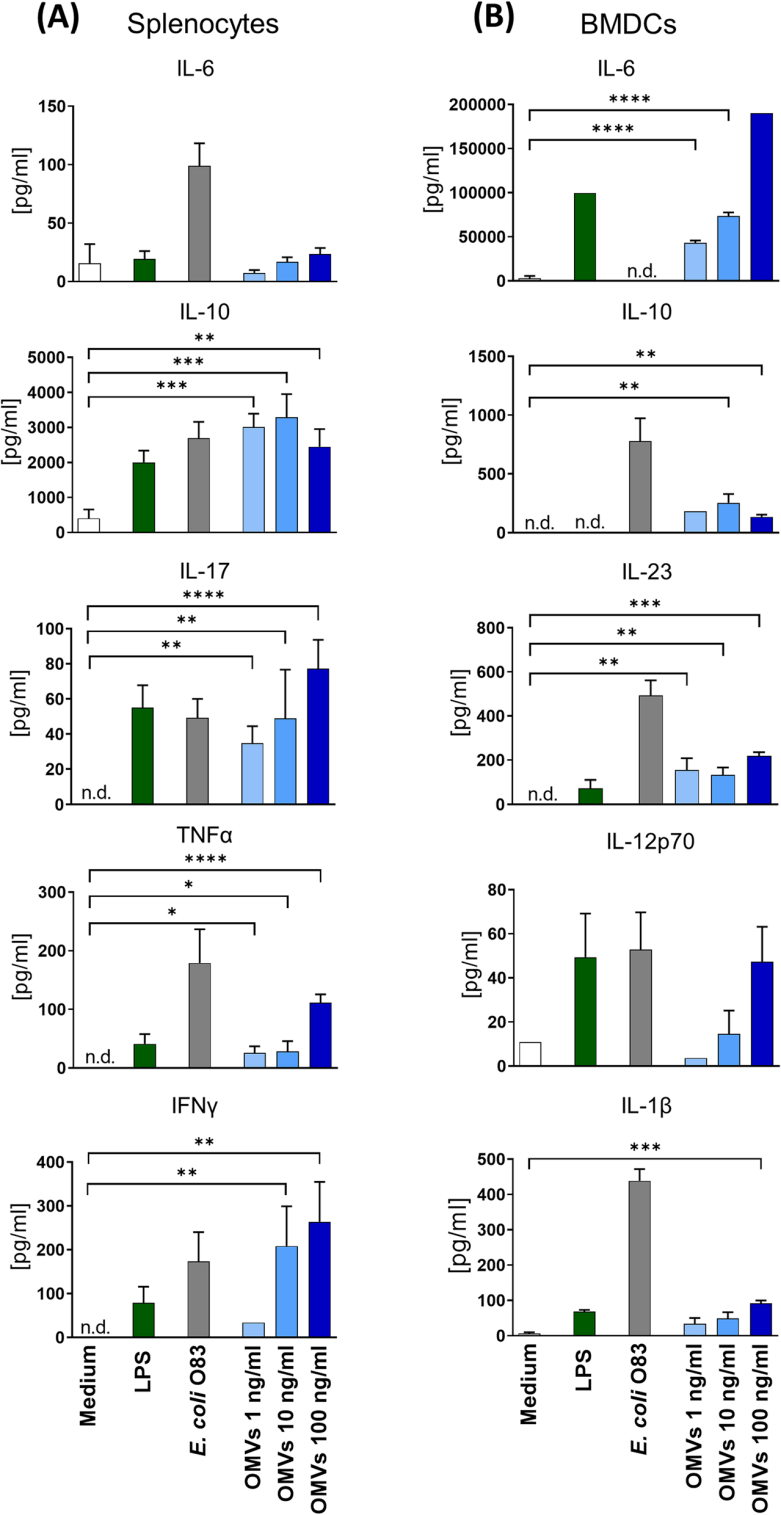
Stimulation of naïve mouse splenocytes and BMDCs with EcO83-OMVs. **A** Mouse splenocytes and (**B**) BMDCs were stimulated with LPS (1 µg/ml), fixed *E. coli* O83 (10^7^ CFU/ml), EcO83-OMVs (OMVs; 1 ng/ml, 10 ng/ml, and 100 ng/ml) for 72 and 48 h, respectively. Unstimulated cells served as controls (Medium). Cytokine levels were measured in the cell culture supernatants by ELISA. Data was analysed using a One-Way ANOVA followed by post-hoc Tukey’s multiple comparison test. *p < 0.5; **p < 0.05; ***p < 0.01. **A** Data are representative of four and (**B**) two independent experiments, respectively. Mean ± SD is shown. Significant differences between EcO83-OMVs and the negative controls are indicated. BMDC = bone marrow-derived dendritic cell; n.d. = not detected

EcO83-OMVs induced high levels of IL-10, IL-17 and IFN-γ in naïve splenocytes in a dose-dependent manner (Fig. 3A). The pro-inflammatory cytokine TNF-α was induced to a much lesser extent and IL-6 was not induced by OMVs at any of the concentrations used (Fig. 3A). EcO83-OMVs are potent inducers of IL-10, a cytokine with regulatory potential, because 1 ng of vesicles induced amounts comparable to the response elicited by 10^7^ CFU/ml fixed *E. coli* O83 bacteria (Fig. 3A). Dendritic cells (DCs) are the most potent antigen-presenting cells and are directly associated with the instruction and regulation of the adaptive immune response [51]. BMDCs stimulated by vesicles produced significant amounts of IL-6, IL-23 and IL-1β (Fig. 3B). The levels of IL-12p70 were not significantly different from those triggered by medium alone (Fig. 3B). To demonstrate that the observed functional effects were associated with EcO83-OMVs and not soluble non-vesicular components, the immunostimulatory effect of EcO83-OMVs was compared with a vesicle-free supernatant and the mock control (Supplementary Material and Methods). Lung cells treated with LPS, *E. coli* O83 and EcO83-OMVs produced IL-6 and TNF-α. No production of these cytokines could be measured in the cultures with vesicle-free supernatant and mock control (Figure S4), ruling out the possibility of a contributing effect of non-vesicular material. We have shown that EcO83-OMVs contain LPS (Table S2) and that they are recognised by TLR4, the key receptor involved in LPS recognition and signal initiation (Fig. 2A). To investigate the role of EcO83-OMVs LPS in immunomodulation, EcO83-OMVs were treated with Polymyxin B or Polymyxin B mock (Supplementary Material and Methods) and then these vesicles were used to treat lung cell cultures. The data show that treatment of EcO83-OMVs with Polymyxin B reduced their ability to trigger the production of IL-6 and TNF-α compared to vesicles treated with Polymyxin mock (Figure S5), suggesting that LPS may be important for the immunostimulatory effect of EcO83-OMVs. Finally, EcO83-OMVs were heat-inactivated at 95 °C for 15 min and added to lung cells (Supplementary Material and Methods) to test whether heat-labile surface molecules are involved in their immunostimulatory properties. The data show that heat inactivation did not reduce the levels of IL-6 and TNF-α in the cell cultures compared to untreated vesicles (Figure S6).

### Preventive effect of intranasally administered EcO83-OMVs on experimental asthma

**Fig. 4:**
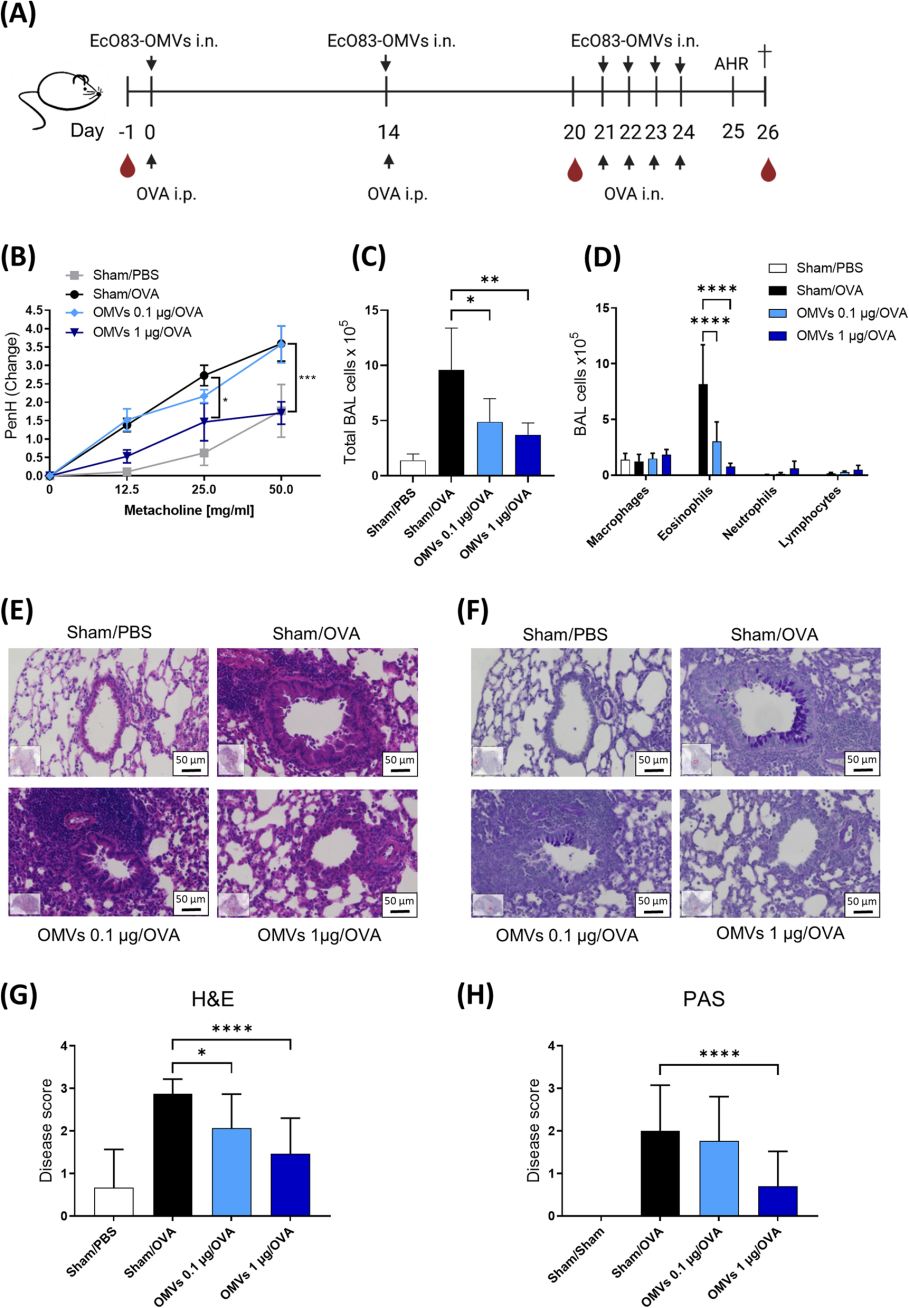
Effect of intranasal application of EcO83-OMVs on the development of allergic airway inflammation. **A** Experimental setup for intranasal application of EcO83-OMVs and induction of experimental allergic airway inflammation to OVA. BALB/c mice were sensitised (i.p.) with either OVA/alum (groups Sham/OVA, OMVs 0.1 µg/OVA and OMVs 1 µg/OVA) or PBS/alum (group Sham/PBS) on days 0 and 14 and challenged (i.n.) with either OVA or PBS on days 21–24 with. Mice were treated i.n. with 30 µl of 0.1 µg EcO83-OMVs (group OMVs 0.1 µg/OVA), 1 µg EcO83-OMVs (group OMVs 1 µg/OVA) or 0.9% NaCl (groups Sham/PBS and Sham/OVA). **B** Airway hyperresponsiveness was measured in response to increasing doses of metacholine by whole body plethysmography. Penh = enhanced pause. **C**-**D** Differential cell counts in bronchoalveolar lavage (BAL): (**C**) Total cell count and (**D**) total numbers of macrophages, eosinophils, neutrophils and lymphocytes. **E**–**F** Representative H&E and PAS-stained lung tissue sections showing recruited inflammatory cells and mucus-producing goblet cells, respectively. **G**-**H** Quantification of histological experiments (Disease score 0–3 for PAS and 1–4 for H&E). Scale bar = 50 µm. **B**, **C**, **E** and **F** Data were analysed using a One-Way ANOVA or (**D**) Two-Way ANOVA followed by post-hoc Tukey’s multiple comparison test. *p < 0.5; **p < 0.05; ***p < 0.01; ****p < 0.001. *n* = 5/group. Data are representative of three independent experiments. Mean ± SD is shown. Significant differences between Sham/OVA and EcO83-OMVs treatment groups (OMVs 0.1 µg/OVA or OMVs 1 µg/OVA) are indicated. alum = aluminiumhydroxide; i.n. = intranasal; i.p. = intraperitoneal; OVA = ovalbumin; OMVs = EcO83-OMVs; H&E = haematoxylin and eosin; PAS = periodic acid-Schiff

In our previous study, intranasal administration of live *E. coli* O83 reduced OVA-induced allergic asthma in a mouse model [17]. Here we tested whether EcO83-OMVs can recapitulate the beneficial effects of live bacteria (Fig. 4A). In this model, repeated exposure of mice to OVA leads to airway hyperresponsiveness, the main feature of asthma (Fig. 4B). Intranasal pretreatment with 1 µg EcO83-OMVs significantly decreased enhanced pause levels after exposure to methacholine compared to sham-treated OVA-sensitised mice, demonstrating the protective effect of vesicles (Fig. 4B).

Intranasal treatment of mice with EcO83-OMVs reduced the total number of cells in the lung (Fig. 4C) and the number of eosinophils in the BAL compared to the sham-treated OVA group (Fig. 4D). The number of macrophages, neutrophils and lymphocytes in the BAL did not change in the EcO83-OMVs group compared to the allergic control group (Fig. 4D). Decreased levels of allergen-specific IgA and IgE in BAL fluid were observed in the OMV-treated mice compared to controls (Figure S7).

**Fig. 5:**
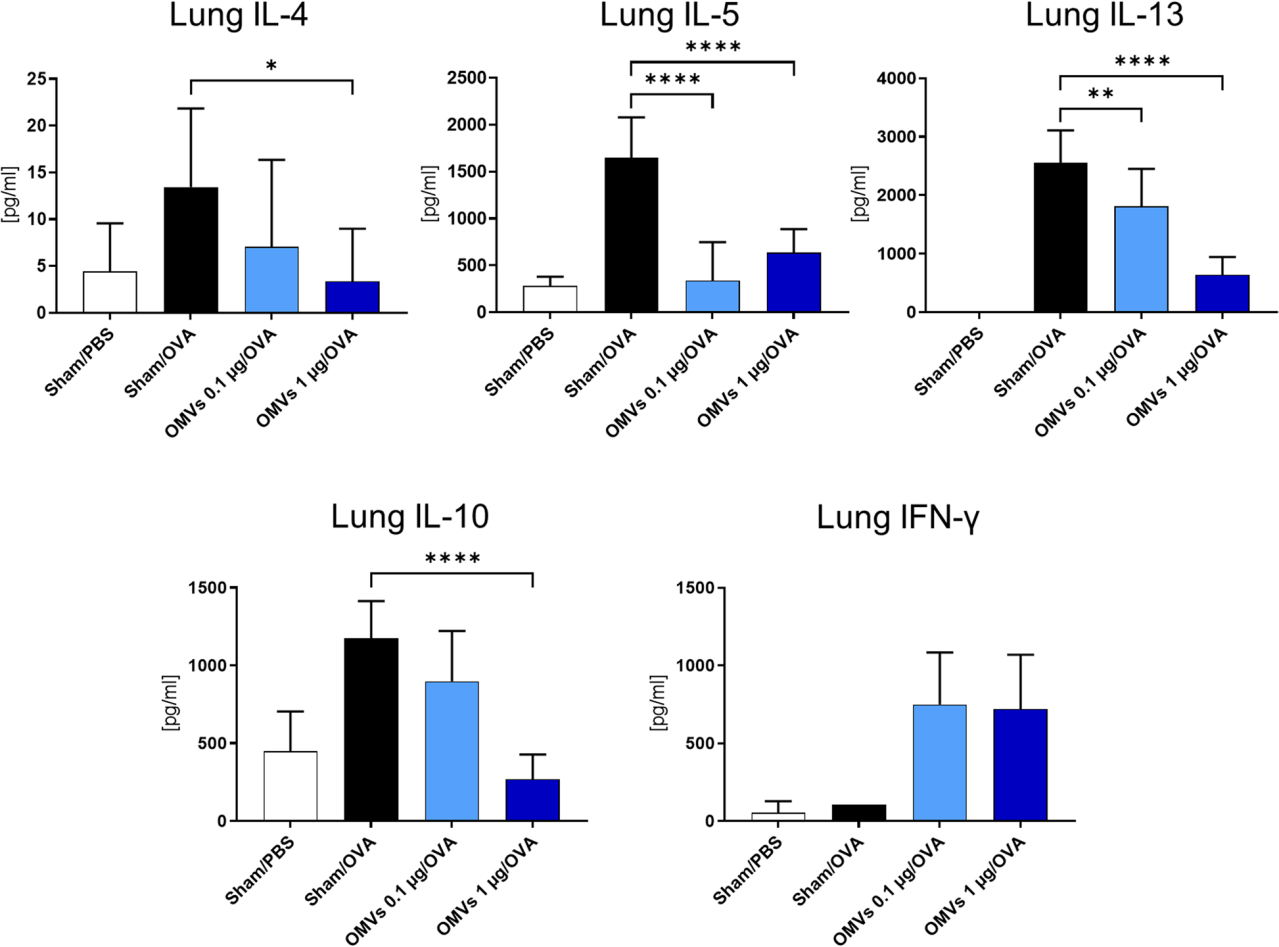
Effect of intranasal application of EcO83-OMVs on the cellular recall response in the lung. Mice were treated as shown in the Fig. 4A. Single cell lung suspensions were stimulated with OVA for 72 h. Cytokines were measured in the supernatant by ELISA. Data were analysed using a One-Way ANOVA followed by post-hoc Tukey’s multiple comparison test. *p < 0.5; **p < 0.05; ****p < 0.001, *n* = 5/group. Data are representative of three independent experiments. Significant differences between sham-treated allergic mice (group Sham/OVA) and EcO83-OMVs treatment groups (OMVs 0.1 µg/OVA or OMVs 1 µg/OVA) are indicated. OVA = ovalbumin; OMVs = EcO83-OMVs

Histological analysis of lung sections confirmed reduced eosinophil infiltration (Fig. 4E) and a decrease in mucus-producing cells (Fig. 4F) in mice treated with 1 µg EcO83-OMVs. This dose also decreased the disease score based on evaluation of H&E-stained samples (Fig. 4G) and mucus production (Fig. 4H). The lover dose of 0.1 µg reduced the disease score based on evaluation of H&E-stained samples (Fig. 4G), but not based on mucus production (Fig. 4H). Single-cell lung suspensions were stimulated with OVA and the levels of IL-4, IL-5, IL-13, IL-10 and IFN-γ were measured in the supernatant. The group treated with 1 µg EcO83-OMVs exhibited significantly lower levels of IL-4, IL-5, IL-13 and IL-10 compared to sham-treated controls (Fig. 5). Treatment with 0.1 µg EcO83-OMVs reduced the levels of IL-5, IL-13 but not IL-4 and IL-10 (Fig. 5) compared to allergic controls. IFN-γ levels were increased in both treatment groups compared to the allergic control, but the effect was not significant (Fig. 5).

The systemic response was assessed by measuring the level of specific antibodies in serum and by the production of cytokines in OVA-restimulated spleen cell cultures. Treatment with EcO83-OMVs had no effect on the levels of OVA-specific serum antibodies (Figure S8A) and OVA-specific IFN-γ in stimulated splenocytes (Figure S8B). We observed a decrease in the levels of IL-5 in OVA-stimulated splenocytes (Figure S8B) after treatment with 1 µg EcO83-OMVs and IL-4, IL-5 and IL-10 after treatment with 0.1 µg EcO83-OMVs compared to sham-treated OVA-sensitised controls.

## Discussion

This is the first study demonstrating a protective effect of OMVs from a probiotic G- bacterium in a mouse model of allergic asthma. Here we describe the isolation, identification, biophysical characterisation, interaction with innate receptors and anti-allergic properties of *E. coli* O83 OMVs.

Although numerous epidemiological studies propose that continuous high exposure to environmental bacteria and endotoxins has a protective effect against allergic sensitisation and asthma, the exact mechanisms are not yet clear [52, 53]. Preclinical studies confirmed the beneficial effect of intranasal administration of farm dust [53, 54] or farm dust microbes [55–57] and suggested that LPS is at least largely responsible for the beneficial effect [53, 54, 58]. We have shown in our previous study that intranasally administered *E. coli* O83 reduced allergy in a TLR4-dependent manner, suggesting the role of LPS [17]. Here we show that EcO83-OMVs, like their parent bacteria contain endotoxin and are recognised by TLR4.

Furthermore, we have shown that treatment of EcO83-OMVs with Polymyxin B (a cyclic cationic polypeptide antibiotic that inhibits LPS-induced TLR4 activation) reduces their ability to trigger the production of IL-6 and TNF-α in lung cells, suggesting that LPS may be important for the immunostimulatory effect of EcO83-OMVs. As the role of LPS displayed on the surface of EcO83-OMVs in the prevention of allergy is tantalising, we are planning a follow-up study using TLR4-defficient mice in an in vivo model of allergic airway inflammation.

The effects of intranasal administration of LPS on pulmonary allergy are complex and to some extent controversial, with some studies showing a worsening, while others show a reduction in allergy [59, 60]. It is now understood that variables such as timing, dosage, chemical structure, and the resulting biological activity of the used LPS can affect the type of immune response that occurs [61]. Therefore, based on its safe and unique properties, EcO83-OMVs have clear advantages over LPS and offer significant potential for a range of clinical applications.

In terms of timing, EcO83-OMVs were administered concomitantly with the allergen, at the time of allergic sensitization and challenge, using the same experimental protocol as we used for the application of live bacteria in our previous study [17]. In this sense, Tulic et al. reported that *Salmonella typhimurium* LPS reduced pulmonary allergy when administered prior or up to 4 days after OVA sensitisation, but worsened it when administered more than 6 days after sensitisation [62]. Furthermore, Bickert et al. have shown that LPS from *E. coli* 026:B6 reduced eosinophilia only when applied at the time of OVA challenge but not when administered before or after sensitisation [63]. Whether prophylactic administration of vesicles prior to sensitisation and challenge has the potential to reduce allergy or whether the beneficial effect is long-lasting remains to be investigated.

It has been demonstrated that the dose of LPS delivered intranasally also determines the direction in which the immune system reacts. A high dose (100 µg) of LPS from a bacterial strain other than *E. coli* O83 reduced allergy to inhaled allergens in mice, but a low dose (0.1 µg) had the reverse effect [64]. Here we show that the higher dose (1 µg) of EcO83-OMVs reduced allergy, whereas a lower dose (0.1 µg) did not worsen the disease severity but improved allergic inflammation in several parameters. Although LPS is a dominant OMV antigen, our results cannot be directly compared with those of Eisenbarth et al*.* also because they used LPS from other bacteria and it is known that LPS can vary greatly between different bacterial strains.

Flagella synthesis has been shown to play a key role in the budding of OMVs from *E. coli* [65]. Here we show that OMVs from *E. coli* O83 contain flagellin (FliC), a major structural protein of bacterial flagellum, and proteins required for anchoring the flagella in the membrane (flagellar hook-associated proteins FlgK, FlgL; hook protein FlgE; basal body rod proteins FlgB, FlgC, FlgE, FlgF; and L-ring protein FlgH). These proteins showed a significant fold enrichment in vesicles compared to the whole bacteria. The cost of constructing flagella is high in bacteria such as *E. coli*, and the reason behind the release of these energy intensive molecules into the environment has yet to be investigated.

Antigen-presenting cells such as DCs recognise flagellin on their apical surface through TLR5, which activates NF-kB and MAPK, leading to the production of pro-inflammatory cytokines [66]. In our studies, EcO83-OMVs, containing high levels of flagellin, induced the expression of pro-inflammatory cytokines such as IL-6 and TNF-α in BMDCs, and the involvement of TLR5 in their recognition was confirmed in TLR5-transfected HEK293 cells. Vesicle-stimulated BMDCs also produce caspase-1-dependent IL-1β, suggesting activation of the NLR neuronal apoptosis inhibitory protein 5 or 6 (NAIP5/6), the sensor for intracellular flagellin, followed by assembly of the NLRC4 inflammasome [67]. However, vesicle-induced IL-1β levels were several 100-fold lower compared to levels induced by whole bacteria, suggesting that vesicles can be viewed as potent immunomodulators that lack some of the negative properties of the parent bacteria. On the other hand, vesicles but not whole bacteria were recognized by TLR5, implying that this receptor is important for vesicles recognition but less important for whole bacteria recognition.

In vivo*,* we have shown that intranasal treatment with vesicles reduces the number of eosinophils in the lungs compared to allergic controls. A similar effect was observed with intranasal application of recombinant flagellin [68, 69]. A recent study suggests that the binding of flagellin to surface receptors on eosinophils such as TLR5 may prevent eosinophil sensitisation [70]. According to Luo et al., flagellin can reduce oxidative stress in eosinophils [69]. We are currently conducting studies to elucidate the effect of EcO83-OMVs on oxidative stress in immune cells.

Recent studies have demonstrated the feasibility of using OMVs to deliver heterogeneous antigens. Eastwood et al. used an innovative expression system in *E. coli* based on a simple peptide tag that results in a high yield of functional proteins packaged into the vesicles [71]. Another study used the “plug-and-play” approach to decorate *S. typhimurium* OMVs with the spike receptor-binding domain [72]. This technology makes it possible to decorate the surface of OMVs with a variety of antigens or even multiple antigens. This could be of interest for the production of engineered OMVs decorated with allergens/peptides for specific immunotherapy. In our previous study, we constructed recombinant *E. coli* Nissle 1917 expressing the chimera with major birch and grass pollen allergenic protein/peptides and showed that its intranasal application prevented poly-sensitization in mice [73]. Therefore, future studies in our laboratory will focus on developing a recombinant *E. coli* O83 to produce OMVs decorated with fluorescent markers or mono- and poly-allergens for specific immunotherapy.

Since there are no studies in which OMVs from different G- bacteria have been tested against allergies, we cannot answer the question of whether it is necessary to use OMVs from probiotic bacteria for allergy prevention or whether OMVs from non-probiotic Gram- bacteria would serve the same purpose. *Neisseria meningitidis*, for example, is a G- pathogenic bacterium and vaccines based on its OMVs have been used in clinics against meningitis [32]. These OMVs contain an extremely potent hexa-acylated LPS that leads to adverse effects. Therefore, several attempts have been made to reduce toxicity by removing the LPS [74]. In summary, we believe that the type of OMVs used depends on the context and purpose of the treatment and that further studies are needed to clarify the effect of OMVs in allergy.

In summary, we show that the probiotic *E. coli* O83 produces OMVs that exhibit potent immunomodulatory and anti-allergic properties. The ease of manufacture and the availability of technologies for genetically engineered vesicles, decorated with heterologous antigens in the lumen or on the surface, provide an extraordinarily versatile platform with great potential for therapeutic applications of bacterial vesicles in allergy research and clinic.

### Supplementary Information

**Additional file 1:** **Figure S1.** Examination of EcO83-OMVs, EcO83-OMVs-depleted supernatant and mock control for bacterial contamination. The isolated EcO83-OMVs, EcO83-OMVs-depleted supernatant and mock control were examined for the presence of live bacteria. Samples were placed on LB agar plates and incubated at 37 °C for 96 h. The plates were examined for visible bacterial colonies. **Figure S2.** Unprocessed image of Bioanalyzer protein analysis of EcO83-OMVs and *E. coli* O83 lysate (modified image in Fig. 1E). Samples 1 and 2: EcO83-OMVs in duplicates. Samples 9 and 10: *E. coli* O83 lysate in duplicates. Samples 3-8: Material not relevant to this publication. **Figure S3.** Activation of the NF-κB pathway by EcO83-OMVs. The macrophage-like mouse cell line RAW264.7 was stimulated with medium, LPS (1 µg/ml), *E. coli* O83 (10^7^ CFU/ml) and EcO83-OMVs (10 ng/ml and 100 ng/ml) at 37 °C and 5 % CO_2_ for 6 h. The expression of IL-6, TNF-α and IL-1β mRNA was measured with RT-PCR and is presented as the fold change to the housekeeping gene GAPDH. OMVs = EcO83-OMVs. **Figure S4.** Immunostimulatory potential of EcO83-OMVs compared to EcO83-OMVs-depleted supernatant and mock control. Cells isolated from the lungs of naive mice (*n* = 5) were treated with medium, LPS (1 µg/ml), *E. coli* O83 (10^8^ CFU/ml), EcO83-OMVs (1 ng/ml, 10 ng/ml, 100 ng/ml), EcO83-OMVs-depleted supernatant and mock control. The supernatant and mock control were added in the amount equal to the volume of vesicles used in cultures with 100 ng/ml EcO83-OMVs and incubated at 37 °C and 5 % CO_2_ for 48 h. IL-6 and TNF-α were measured in the cell culture supernatant by ELISA. The mean ± SD is shown. Data were analysed using a One-Way ANOVA followed by a post-hoc Tukey’s multiple comparison test. **p<0.05; ****p<0.001. OMVs = EcO83-OMVs. **Figure S5.** Immunostimulatory potential of EcO83-OMVs compared to Polymyxin B-treated EcO83-OMVs. Cells isolated from the lungs of naive mice (*n* = 5) were treated with medium, LPS (1 µg/ml), *E. coli* O83 (10^8^ CFU/ml), EcO83-OMVs treated with Polymyxin B (1 ng/ml, 10 ng/ml, 100 ng/ml), EcO83-OMVs treated with Polymyxin B mock (1 ng/ml, 10 ng/ml, 100 ng/ml) and stimulated with Polymyxin B alone at 37 °C/ 5% CO_2_ for 48 h. IL-6 and TNF-α were measured in the supernatants by ELISA. The mean ± SD is shown. Data were analysed using a One-Way ANOVA followed by a post-hoc Tukey’s multiple comparison test. ***p < 0.01; ****p < 0.001. OMVs = EcO83-OMVs ; PmB = Polymyxin B. **Figure S6.** Immunostimulatory potential of EcO83-OMVs compared to heat-treated EcO83-OMVs. Cells isolated from the lungs of naive mice (*n* = 5) were treated with medium, LPS (1 µg/ml), *E. coli* O83 (10^8^ CFU/ml), EcO83-OMVs (1 ng/ml, 10 ng/ml, 100 ng/ml) and heat-treated EcO83-OMVs (1 ng/ml, 10 ng/ml, 100 ng/ml) at 37 °C/ 5 % CO_2_ for 48 h. IL-6 and TNF-α were measured in the supernatants by ELISA. The mean ± SD is shown. Data were analysed using a One-Way ANOVA followed by a post-hoc Tukey’s multiple comparison test. ****p<0.001. OMVs = EcO83-OMVs. **Figure S7.** Local allergen-specific responses: Levels of OVA-specific antibodies in BAL. BAL was collected on sacrifice day by flushing the lungs with PBS and OVA-specific antibodies were measured by ELISA. Data were analysed using a One-Way ANOVA followed by post-hoc Tukey’s multiple comparison test. *p<0.5; **p<0.05. *n* = 5/group. Data are representative of three independent experiments. Mean ± SD is shown. Significant differences between Sham/OVA and EcO83-OMVs treatment groups (OMVs 0.1 µg/OVA or OMVs 1 µg/OVA) are indicated. BAL = bronchoalveolar lavage; OVA = ovalbumin; OMVs = EcO83-OMVs. **Figure S8.** Systemic allergen-specific responses: Levels of OVA-specific antibodies in serum and cytokines produced by OVA-stimulated splenocytes* ex vivo.* (A) Blood was sampled on days -1, 20 and 26 (Experimental setup see Figure 5 A) and centrifuged to obtain serum. OVA-specific antibodies in serum were measured by ELISA. (B) Single cell suspensions of excised spleens of Sham or EcO83-OMVs-treated allergic mice were stimulated with OVA for 72 h and cytokine levels were measured by ELISA. Data was analysed using a One-Way ANOVA followed by post-hoc Tukey’s multiple comparison test. *p<0.5; **p<0.05; ***p<0.001. (A) Data are representative of three independent experiments for samples collected on day -1 and a pool of two experiments for samples collected on days 20 and 26; *n* = 5-10/group. (**B**) Data are representative of three independent experiments, *n* = 5/group. Mean ± SD is shown. Significant differences between Sham/OVA and EcO83-OMVs treatment groups (OMVs 0.1 µg/OVA or OMVs 1 µg/OVA) are indicated. OVA = ovalbumin; OMVs = EcO83-OMVs. **Table S1.** Cell culture media. **Table S2.** Size distribution and concentration of vesicles and amount of protein, and endotoxin corresponding to material collected from a two litre culture. Average ± SD is shown from three independent measurements. **Table S3.** Protein content in EcO83-OMVs measured with Bioanalyzer. Marked in grey are dominant visible bands. **Table S4.** List of proteins that were found to be upregulated in EcO83-OMVs compared to *E. coli* O83 lysate and met the false discovery rate (FDR) criteria. (P-value<0.05). n.d. = not determined.

## Data Availability

The mass spectrometry proteomics data have been deposited to the ProteomeXchange Consortium via the PRIDE partner repository (http://www.ebi.ac.uk/pride) with the dataset identifier PXD040963.

## References

[1] Akdis CA (2021). Does the epithelial barrier hypothesis explain the increase in allergy, autoimmunity and other chronic conditions?. Nat Rev Immunol.

[2] Dorofeeva Y, Shilovskiy I, Tulaeva I, Focke-Tejkl M, Flicker S, Kudlay D (2021). Past, present, and future of allergen immunotherapy vaccines. Allergy: Europ J Allergy Clinical Immunol.

[3] Campbell DE, Boyle RJ, Thornton CA, Prescott SL (2015). Mechanisms of allergic disease - environmental and genetic determinants for the development of allergy. Clinical Experiment Allergy.

[4] Sokolowska M, Frei R, Lunjani N, Akdis CA, O’Mahony L (2018). Microbiome and asthma. Asthma Res Pract.

[5] Jatzlauk G, Bartel S, Heine H, Schloter M, Krauss-Etschmann S (2017). Influences of environmental bacteria and their metabolites on allergies, asthma, and host microbiota. Allergy: Europ J Allergy Clinical Immunol.

[6] Lopez-Santamarina A, Gonzalez EG, Lamas A, del Mondragon AC, Regal P, Miranda JM (2021). Probiotics as a possible strategy for the prevention and treatment of allergies A narrative review. Foods.

[7] Cao L, Wang L, Yang L, Tao S, Xia R, Fan W (2015). Long-term effect of early-life supplementation with probiotics on preventing atopic dermatitis: A meta-analysis. J Dermatolog Treatment.

[8] Panduru M, Panduru NM, Sələvəstru CM, Tiplica GS (2015). Probiotics and primary prevention of atopic dermatitis: A meta-analysis of randomized controlled studies. J Europ Academ Dermatology Venereol.

[9] Abrahamsson TR, Jakobsson T, Björkstén B, Oldaeus G, Jenmalm MC (2013). No effect of probiotics on respiratory allergies: A seven-year follow-up of a randomized controlled trial in infancy. Pediatric Allergy Immunol.

[10] Azad MB, Coneys GJ, Kozyrskyj AL, Field CJ, Ramsey CD, Becker AB (2013). Probiotic supplementation during pregnancy or infancy for the prevention of asthma and wheeze Systematic review and meta-Analysis. BMJ (Online)..

[11] Wei X, Jiang P, Liu J, Sun R, Zhu L (2020). Association between probiotic supplementation and asthma incidence in infants: a meta-analysis of randomized controlled trials. J Asthma.

[12] Pellaton C, Nutten S, Thierry A-C, Boudousquié C, Barbier N, Blanchard C (2012). Intragastric and intranasal administration of lactobacillus paracasei NCC2461 modulates allergic airway inflammation in mice. Int J Inflam..

[13] Spacova I, Petrova MI, Fremau A, Pollaris L, Vanoirbeek J, Ceuppens JL (2019). Intranasal administration of probiotic Lactobacillus Rhamnosus GG prevents birch pollen-induced allergic asthma in a murine model. Allergy: Europ J Allergy Clinical Immunol.

[14] Schabussova I, Hufnagl K, Wild C, Nutten S, Zuercher AW, Mercenier A (2011). Distinctive anti-allergy properties of two probiotic bacterial strains in a mouse model of allergic poly-sensitization. Vaccine.

[15] Lodinova-Zadnikova R, Prokešová L, Kocourková I, Hrdý J, Žižka J (2010). Prevention of allergy in infants of allergic mothers by probiotic escherichia coli. Int Arch Allergy Immunol..

[16] Lodinova-Zadnikova R, Cukrowska B, Tlaskalova-Hogenova H (2003). Oral administration of probiotic Escherichia coli after birth reduces frequency of allergies and repeated infections later in life (after 10 and 20 years). Int Arch Allergy Immunol..

[17] Zwicker C, Sarate P, Drinić M, Ambroz K, Korb E, Smole U (2018). Prophylactic and therapeutic inhibition of allergic airway inflammation by probiotic Escherichia coli O83. J Allergy Clinical Immunol.

[18] Pradhan D, Mallappa RH, Grover S (2020). Comprehensive approaches for assessing the safety of probiotic bacteria. Food Control.

[19] Kothari D, Patel S, Kim SK (2019). Probiotic supplements might not be universally-effective and safe: a review. Biomedic Pharmacother.

[20] Schwechheimer C, Kuehn MJ (2015). Outer-membrane vesicles from Gram-negative bacteria: Biogenesis and functions. Nat Rev Microbiol.

[21] Hu R, Li J, Zhao Y, Lin H, Liang L, Wang M (2020). Exploiting bacterial outer membrane vesicles as a cross-protective vaccine candidate against avian pathogenic Escherichia coli (APEC). Microb Cell Fact.

[22] David L, Bordignon P, Meunier E, Oswald E (2021). Outer membrane vesicles produced by pathogenic strains of Escherichia coli block autophagic flux and exacerbate inflammasome activation. Autophagy.

[23] Bielaszewska M, Marejková M, Bauwens A, Kunsmann-Prokscha L, Mellmann A, Karch H (2018). Enterohemorrhagic Escherichia coli O157 outer membrane vesicles induce interleukin 8 production in human intestinal epithelial cells by signaling via Toll-like receptors TLR4 and TLR5 and activation of the nuclear factor NF-κB. Int J Medical Microbiol.

[24] Bielaszewska M, Rüter C, Bauwens A, Greune L, Jarosch K-A, Steil D, et al. Host cell interactions of outer membrane vesicle- associated virulence factors of enterohemorrhagic Escherichia coliO157: Intracellular delivery, trafficking and mechanisms of cell injury. PLoS Pathog. 2017;13(2):e1006159. 10.1371/journal.ppat.1006159 PMC5310930 28158302

[25] Hu R, Lin H, Li J, Zhao Y, Wang M, Sun X (2020). Probiotic Escherichia coli Nissle 1917-derived outer membrane vesicles enhance immunomodulation and antimicrobial activity in RAW264.7 macrophages. BMC Microbiol.

[26] Cañas MA, Giménez R, Fábrega MJ, Toloza L, Baldomà L, Badia J (2016). Outer membrane vesicles from the probiotic Escherichia coli Nissle 1917 and the commensal ECOR12 enter intestinal epithelial cells via clathrin-dependent endocytosis and elicit differential effects on DNA damage. PLoS One..

[27] Fábrega MJ, Rodríguez-Nogales A, Garrido-Mesa J, Algieri F, Badía J, Giménez R (2017). Intestinal anti-inflammatory effects of outer membrane vesicles from Escherichia coli Nissle 1917 in DSS-experimental colitis in mice. Front Microbiol.

[28] Diaz-Garrido N, Badia J, Baldoma L. Microbiota-derived extracellular vesicles in interkingdom communication in the gut. J Extracell Vesicles. 2021;10(13):e12161. 10.1002/jev2.12161 PMC8568775 34738337

[29] Kaparakis-Liaskos M, Ferrero RL (2015). Immune modulation by bacterial outer membrane vesicles. Nat Rev Immunol.

[30] Bitto NJ, Kaparakis-Liaskos M (2017). The therapeutic benefit of bacterial membrane vesicles. Int J Mol Sci..

[31] Gilmore WJ, Johnston EL, Zavan L, Bitto NJ, Kaparakis-Liaskos M (2021). Immunomodulatory roles and novel applications of bacterial membrane vesicles. Mol Immunol..

[32] Ladhani SN, Ramsay M, Borrow R, Riordan A, Watson JM, Pollard AJ (2016). Enter B and W: Two new meningococcal vaccine programmes launched. Arch Dis Child..

[33] Kohl P, Zingl FG, Eichmann TO, Schild S. Isolation of outer membrane vesicles including their quantitative and qualitative analyses. Methods Mol Biol. 2018;1839:117–34. 10.1007/978-1-4939-8685-9_11 30047059

[34] Edhager AV, Povlsen JA, Løfgren B, Bøtker HE, Palmfeldt J (2018). Proteomics of the Rat Myocardium during Development of Type 2 Diabetes Mellitus Reveals Progressive Alterations in Major Metabolic Pathways. J Proteome Res..

[35] Cox J, Mann M (2008). MaxQuant enables high peptide identification rates, individualized p.p.b.-range mass accuracies and proteome-wide protein quantification. Nat Biotechnol.

[36] Tyanova S, Temu T, Sinitcyn P, Carlson A, Hein MY, Geiger T (2016). The Perseus computational platform for comprehensive analysis of (prote)omics data. Nat Methods..

[37] Goedhart J, Luijsterburg MS (2020). VolcaNoseR is a web app for creating, exploring, labeling and sharing volcano plots. Sci Rep..

[38] Perez-Riverol Y, Bai J, Bandla C, García-Seisdedos D, Hewapathirana S, Kamatchinathan S (2022). The PRIDE database resources in 2022: a hub for mass spectrometry-based proteomics evidences. Nucleic Acids Res..

[39] Srutkova D, Schwarzer M, Hudcovic T, Zakostelska Z, Drab V, Spanova A, et al. Bifidobacterium longum CCM 7952 Promotes Epithelial Barrier Function and Prevents Acute DSS- Induced Colitis in Strictly Strain-Specific Manner. PLoS ONE. 2015;10(7):e0134050. 10.1371/journal.pone.0134050 PMC4517903 26218526

[40] Korb E, Drinić M, Wagner A, Geissler N, Inic-Kanada A, Peschke R (2021). Reduction of allergic lung disease by mucosal application of toxoplasma Gondii-Derived molecules: possible role of carbohydrates. Front Immunol.

[41] Drinic M, Wagner A, Sarate P, Zwicker C, Korb E, Loupal G, et al. Toxoplasma gondii tachyzoite-extract acts as a potent immunomodulator against allergic sensitization and airway inflammation. Sci Rep. 2017;7(1):15211. 10.1038/s41598-017-15663-4 PMC5680314 29123241

[42] Zaiss MM, Rapin A, Lebon L, Dubey LK, Mosconi I, Sarter K (2015). The intestinal microbiota contributes to the ability of helminths to modulate allergic inflammation. Immunity.

[43] Schoenhals G, Whitfield C (1993). Comparative analysis of flagellin sequences from Escherichia coli strains possessing serologically distinct flagellar filaments with a shared complex surface pattern. J Bacteriol.

[44] Takeuchi O, Hoshino K, Kawai T, Sanjo H, Takada H, Ogawa T (1999). Differential roles of TLR2 and TLR4 in recognition of Gram-Negative and Gram-Positive bacterial cell wall components. Technol Japan Scie.

[45] Hayashi F, Smith KD, Ozinsky A, Hawn TR, Yi EC, Goodlett DR (2001). The innate immune response to bacterial flagellin is mediated by Toll-like receptor 5. Nature..

[46] Cecil JD, O’Brien-Simpson NM, Lenzo JC, Holden JA, Chen YY, Singleton W (2016). Differential responses of pattern recognition receptors to outer membrane vesicles of three periodontal pathogens. PLoS One..

[47] Kaparakis M, Turnbull L, Carneiro L, Firth S, Coleman HA, Parkington HC (2010). Bacterial membrane vesicles deliver peptidoglycan to NOD1 in epithelial cells. Cell Microbiol.

[48] Ou T, Lilly M, Jiang W. The Pathologic Role of Toll-Like Receptor 4 in Prostate Cancer. Front Immunol. 2018;9:1188. 10.3389/fimmu.2018.01188 PMC5998742 29928275

[49] Cochet F, Peri F. The Role of Carbohydrates in the Lipopolysaccharide (LPS)/Toll-Like Receptor 4 (TLR4) Signalling. Int J Mol Sci. 2017;18(11):2318. 10.3390/ijms18112318 PMC5713287 29099761

[50] Liu T, Zhang L, Joo D, Sun S-C (2017). NF-κB signaling in inflammation. Signal Transduct Target Ther..

[51] Schuijs MJ, Hammad H, Lambrecht BN (2019). Professional and ‘Amateur’ Antigen-Presenting Cells In Type 2 Immunity. Trends Immunol.

[52] Ege M, Mayer M, Normand AC, Genuneit J, Cookson W, Braun-Fahrländer Ch (2011). Exposure to environmental microorganisms and childhood asthma. N Engl J Med.

[53] Stein MM, Hrusch CL, Gozdz J, Igartua C, Pivniouk V, Murray SE (2016). Innate Immunity and Asthma Risk in Amish and Hutterite farm children. New England Medicine.

[54] Schuijs MJ, Willart MA, Vergote K, Gras D, Deswarte K, Ege MJ (1979). Farm dust and endotoxin protect against allergy through A20 induction in lung epithelial cells. Science.

[55] Debarry J, Garn H, Hanuszkiewicz A, Dickgreber N, Blumer N, von Mutius E (2007). Acinetobacter lwoffii and Lactococcus lactis strains isolated from farm cowsheds possess strong allergy-protective properties. J Allergy Clin Immunol..

[56] Hagner S, Harb H, Zhao M, Stein K, Holst O, Ege MJ (2013). Farm-derived Gram-positive bacterium Staphylococcus sciuri W620 prevents asthma phenotype in HDM- and OVA-exposed mice. Allergy: Europ J Allergy Clinical Immunol.

[57] Conrad ML, Ferstl R, Teich R, Brand S, Blumer N, Yildirim AO (2009). Maternal TLR signaling is required for prenatal asthma protection by the nonpathogenic microbe Acinetobacter lwoffii F78. J Exp Med..

[58] Debarry J, Hanuszkiewicz A, Stein K, Holst O, Heine H (2010). The allergy-protective properties of Acinetobacter lwoffii F78 are imparted by its lipopolysaccharide. Allergy: Europ J Allergy Clinical Immunol.

[59] Murakami D, Yamada H, Yajima T, Masuda A, Komune S, Yoshikai Y (2007). Lipopolysaccharide inhalation exacerbates allergic airway inflammation by activating mast cells and promoting Th2 responses. Clinical Experimental Allergy.

[60] Blumer N, Herz U, Wegmann M, Renz H, Blümer N, Herz U (2005). Prenatal lipopolysaccharide-exposure prevents allergic sensitization and airway inflammation, but not airway responsiveness in a murine model of experimental asthma. Clinical Experiment Allergy.

[61] Liu AH (2002). Endotoxin exposure in allergy and asthma: Reconciling a paradox. J Allergy Clinical Immunol.

[62] Tulić MK, Wale JL, Holt PG, Sly PD (2000). Modification of the inflammatory response to allergen challenge after exposure to bacterial lipopolysaccharide. Am J Respir Cell Mol Biol..

[63] Bickert T, Trujillo-Vargas CM, Duechs M, Wohlleben G, Polte T, Hansen G (2009). Probiotic Escherichia coli Nissle 1917 suppresses Allergen-Induced Th2 responses in the Airways. Int Arch Allergy Immunol.

[64] Eisenbarth SC, Piggott DA, Huleatt JW, Visintin I, Herrick CA, Bottomly K (2002). Lipopolysaccharide-enhanced, toll-like receptor 4-dependent T helper cell type 2 responses to inhaled antigen. J Experimental Medicine.

[65] Manabe T, Kato M, Ueno T, Kawasaki K (2013). Flagella proteins contribute to the production of outer membrane vesicles from Escherichia coli W3110. Biochem Biophys Res Commun.

[66] Feuillet V, Medjane S, Mondor I, Demaria O, Pagni PP, Galán JE (2006). Involvement of toll-like receptor 5 in the recognition of flagellated bacteria. Proc Natl Acad Sci U S A..

[67] Zhong M, Yan H, Li Y (2017). Flagellin: A unique microbe-associated molecular pattern and a multi-faceted immunomodulator. Cell Mol Immunol..

[68] Lee DH, Park HK, Lee HR, Sohn H, Sim S, Park HJ (2022). Immunoregulatory effects of Lactococcus lactis-derived extracellular vesicles in allergic asthma. Clin Transl Allergy..

[69] Luo XQ, Liu J, Mo LH, Yang G, Ma F, Ning Y (2021). Flagellin alleviates airway allergic response by stabilizing eosinophils through modulating oxidative stress. J Innate Immun.

[70] Lv X, Chang Q, Wang Q, Jin QR, Liu HZ, Yang SB (2022). Flagellin maintains eosinophils in the intestine. Cytokine..

[71] Eastwood TA, Baker K, Streather BR, Hiscock JR, Lennon C, Mulvihill DP (2023). High-yield vesicle-packaged recombinant protein production from E coli. Cell Reports Methods.

[72] Jiang L, Driedonks TAP, Jong WSP, Dhakal S, Bart van den Berg van Saparoea H, Sitaras I, et al. A bacterial extracellular vesicle-based intranasal vaccine against SARS-CoV-2 protects against disease and elicits neutralizing antibodies to wild-type and Delta variants. J Extracell Vesicles. 2022;11. 10.1002/jev2.12192 PMC8920961 35289114

[73] Sarate PJ, Heinl S, Poiret S, Drinić M, Zwicker C, SchabussovaE. coli Nissle,  I (1917). is a safe mucosal delivery vector for a birch-grass pollen chimera to prevent allergic poly-sensitization. Mucosal Immunol..

[74] Afshin Z, Joep B, de Bas W, Jan HH, Bth E, Elly R (2016). Meningococcal Outer Membrane Vesicle Composition-Dependent Activation of the Innate Immune Response. Infect Immun.

