# Correction: Extracellular vesicles of the probiotic bacteria *E. coli* O83 activate innate immunity and prevent allergy in mice

**DOI:** 10.1186/s12964-023-01405-9

**Published:** 2023-12-06

**Authors:** Anna Marlene Schmid, Agnieszka Razim, Magdalena Wysmołek, Daniela Kerekes, Melissa Haunstetter, Paul Kohl, Georgii Brazhnikov, Nora Geissler, Michael Thaler, Eliška Krčmářová, Martin Šindelář, Tamara Weinmayer, Jiří Hrdý, Katy Schmidt, Peter Nejsum, Bradley Whitehead, Johan Palmfeldt, Stefan Schild, Aleksandra Inić-Kanada, Ursula Wiedermann, Irma Schabussova

**Affiliations:** 1 Institute of Specifc Prophylaxis and Tropical Medicine, Center for Pathophysiology, Infectiology and Immunology, Medical University of Vienna, 1090 Vienna, Austria; 2 Hirszfeld Institute of Immunology and Experimental Therapy, Polish Academy of Sciences, Wroclaw, Poland; 3 Institute of Molecular Biosciences, Karl-Franzens-University, Graz, Austria; 4 Institute of Immunology and Microbiology, First Faculty of Medicine, Charles University, and General University Hospital, Prague, Czech Republic; 5 Department of Experimental Biology, Faculty of Science, Masaryk University, Brno, Czech Republic; 6 Core Facility for Cell Imaging and Ultrastructural Research, Faculty of Life Sciences, University of Vienna, Vienna, Austria; 7 Department of Infectious Diseases, Aarhus University Hospital, Aarhus, Denmark; 8 Department of Clinical Medicine, Aarhus University, Aarhus, Denmark; 9 BioTechMed, Graz, Austria; 10 Field of Excellence Biohealth – University of Graz, Graz, Austria

**Correction: Cell Commun Signal 21, 297 (2023)**

**https://doi.org/10.1186/s12964-023-01329-4**

Following publication of the original article [1], the authors reported that the authors’ contributions had been mentioned in the funding section instead of the funding note.

The funding note should read: This project was funded by the Austrian Science Fund (FWF), projects P 34867 (I.S., M.W. and M.T.), P 33073 (S.S.), and the PhD Program Molecular, Cellular and Clinical Allergology DK W1248-B30 (N.G., G.B. and U.W.); the Government of Lower Austria Danube Allergy Research Cluster (D.K., G.B., N.G., T.W., A.I.K., U.W., and I.S.); BioTechMed-Graz Flagship Project Secretome (S.S.); the OeAD WTZ grants CZ 06/2020 (A.S. and I.S.), PL 03/2022 (I.S.), RS 08/2022 (A.S. and I.S.), PL 04/2019 (I.S.), and CZ 07/2023 (I.S. and J.H.); and by European Union through MSCA-PF project LactoVES (Grant Agreement no. 101066450 (A.R.).

Additional file 1 has also been correctly linked to the original article.

The original article[1] has been corrected.
