# Supplementary material for: Extracellular vesicles of the probiotic bacteria E. coli O83 activate innate immunity and prevent allergy in mice
Source: Cell Commun Signal. 2023 Oct 20;21:297. doi: 10.1186/s12964-023-01329-4 (PMC10588034; doi:10.1186/s12964-023-01329-4)
Supplement: Supplementary file 2 — Additional file 1: Figure S1. Examination of EcO83-OMVs, EcO83-OMVs-depleted supernatant and mock control for bacterial contamination. The isolated EcO83-OMVs, EcO83-OMVs-depleted supernatant and mock control were examined for the presence of live bacteria. Samples were placed on LB agar plates and incubated at 37 °C for 96 h. The plates were examined for visible bacterial colonies. Figure S2. Unprocessed image of Bioanalyzer protein analysis of EcO83-OMVs and E. coli O83 lysate (modified image in Fig. 1E). Samples 1 and 2: EcO83-OMVs in duplicates. Samples 9 and 10: E. coli O83 lysate in duplicates. Samples 3-8: Material not relevant to this publication. Figure S3. Activation of the NF-κB pathway by EcO83-OMVs. The macrophage-like mouse cell line RAW264.7 was stimulated with medium, LPS (1 µg/ml), E. coli O83 (107 CFU/ml) and EcO83-OMVs (10 ng/ml and 100 ng/ml) at 37 °C and 5 % CO2 for 6 h. The expression of IL-6, TNF-α and IL-1β mRNA was measured with RT-PCR and is presented as the fold change to the housekeeping gene GAPDH. OMVs = EcO83-OMVs. Figure S4. Immunostimulatory potential of EcO83-OMVs compared to EcO83-OMVs-depleted supernatant and mock control. Cells isolated from the lungs of naive mice (n = 5) were treated with medium, LPS (1 µg/ml), E. coli O83 (108 CFU/ml), EcO83-OMVs (1 ng/ml, 10 ng/ml, 100 ng/ml), EcO83-OMVs-depleted supernatant and mock control. The supernatant and mock control were added in the amount equal to the volume of vesicles used in cultures with 100 ng/ml EcO83-OMVs and incubated at 37 °C and 5 % CO2 for 48 h. IL-6 and TNF-α were measured in the cell culture supernatant by ELISA. The mean ± SD is shown. Data were analysed using a One-Way ANOVA followed by a post-hoc Tukey’s multiple comparison test. **p<0.05; ****p<0.001. OMVs = EcO83-OMVs. Figure S5. Immunostimulatory potential of EcO83-OMVs compared to Polymyxin B-treated EcO83-OMVs. Cells isolated from the lungs of naive mice (n = 5) were treated with medium, LPS (1 µg/ml), E. coli [file 12964_2023_1329_MOESM1_ESM.docx]

**SUPPLEMENTARY MATERIAL**

**Title:** Extracellular vesicles of the probiotic bacteria *E. coli* O83 activate innate immunity and prevent allergy in mice

**Running title:** Extracellular vesicles of *E. coli* O83 reduce allergy

**Authors:** Anna Marlene Schmid^1^, Agnieszka Razim^1,2^, Magdalena Wysmołek^1^, Daniela Kerekes^1^, Melissa Haunstetter^1^, Paul Kohl^3^, Georgii Brazhnikov^1^, Nora Geissler^1^, Michael Thaler^1^, Eliška Krčmářová^4^, Martin Šindelář^5^, Tamara Weinmayer^1^, Jiří Hrdý^4^, Katy Schmidt^6,^°, Peter Nejsum^7,8^, Bradley Whitehead^7,8^, Johan Palmfeldt^8^, Stefan Schild^3,9,10^, Aleksandra Inić -Kanada^1^, Ursula Wiedermann^1^ and Irma Schabussova^1^

^1^Institute of Specific Prophylaxis and Tropical Medicine, Center for Pathophysiology,

Infectiology and Immunology, Medical University of Vienna, Vienna, Austria

^2^Hirszfeld Institute of Immunology and Experimental Therapy, Polish Academy of

Sciences, Wroclaw, Poland

^3^Institute of Molecular Biosciences, Karl-Franzens-University, Graz, Austria

^4^Institute of Immunology and Microbiology, First Faculty of Medicine, Charles University, and General University Hospital, Prague, Czech Republic

^5^Department of Experimental Biology, Faculty of Science, Masaryk University,

Brno, Czech Republic

^6^Center for Anatomy and Cell Biology, Medical University of Vienna, Vienna, Austria

^7^Department of Infectious Diseases, Aarhus University Hospital, Aarhus, Denmark

^8^Department of Clinical Medicine, Aarhus University, Aarhus, Denmark

^9^BioTechMed, Graz, Austria

^10^Field of Excellence Biohealth – University of Graz, Graz, Austria

**Current affiliation:**

°Core facility for Cell Imaging and Ultrastructural Research, Faculty of Life Sciences,

University of Vienna

**Correspondence:**

Assoc.-Prof. Univ.-Doc. Irma Schabussova, PhD

Institute of Specific Prophylaxis and Tropical Medicine

Medical University of Vienna

Kinderspitalgasse 15, 1090 Vienna, Austria

Email: irma.schabussova@meduniwien.ac.at

**SUPPLEMENTARY MATERIALS AND METHODS**

**Table S1: Cell culture media**

| **Purpose of media** | **Media and additives** | **Supplier** |
| --- | --- | --- |
| HEK-293/hTLR2 | Dulbecco’s modified Eagle’s medium (DMEM)  Heat inactivated fetal calf serum (FCS) 10 %  Penicillin/streptomycin (Pen/Strep)  50 U/ml/ 50 µg/ml  Blasticidin 10 µg/ml | Gibco   Gibco  Biochrom  Invivogen |
| HEK-293/hTLR4/CD14/MD2 | DMEM  FCS 10 %  Pen/Strep 50 U/ml/ 50 µg/ml  Blasticidin 10 µg/ml  Hygromycin B Gold 55 µg/ml | Gibco  Gibco  Biochrom  Invivogen  Invivogen |
| HEK-293/hTLR5 | DMEM  FCS 10 %  Pen/Strep 50 U/ml/ 50 µg/ml  Normocin 100 µg/ml  L-glutamine 2 mM  Blasticidin 10 µg/ml | Gibco  Gibco  Biochrom  Invivogen  Gibco  Invivogen |
| HEK-293/hNOD1  HEK-293/hNOD2 | DMEM  FCS 10 %  Blasticidin 10 µg/ml  Normocin 110 µg/ml | Gibco  Gibco  Invivogen  Invivogen |
| Complete RPMI | RPMI 1640  FCS 10 %  Mercaptoethanol 2 mM  L-glutamine 2 mM  Gentamycin 100 µg/ml | Biowest  Gibco  Sigma Aldrich  Sigma Aldrich  Carl Roth |
| Wash RPMI | RPMI 1640  Gentamycin 100 µg/ml | Biowest  Carl Roth |
| Bone marrow-derived dendritic cells | RPMI 1640  FCS 10 %  L-glutamine 2 mM  Pen/Strep 50 U/ml/ 50 µg/ml | Biowest  Gibco  Sigma Aldrich  Biochrom |

Isolation and restimulation of spleen cells *ex vivo*

Spleens from EcO83-treated OVA-allergic mice were processed as described in the main text. Cells were seeded at a concentration of 5 x 10^6^ cells/ml and stimulated with endotoxin-free OVA (100 µg/ml; EndoGrade; Hyglos, Bernried am Starnberger See, Germany) for 72 h at 37 °C and 5 % CO_2_. Cell culture supernatants were collected and cytokines were analysed by ELISA.

**ELISA**

To measure OVA-specific antibodies, an in-house established ELISA protocol was used as described before [1].

**Preparation of a mock control and recovery of the EcO83-OMVs-depleted supernatant**

To demonstrate that the immunomodulatory effects were related to EcO83-OMVs and not soluble molecules, we tested two different controls. For the mock control, the BHI medium was incubated for 8 h at 37 °C and 200 rpm without the addition of bacteria. The medium was processed in parallel with the bacterial culture (described in Materials and Methods). To obtain the fraction depleted of EcO83-OMVs, the bacterial culture supernatant was centrifuged, filtered and ultracentrifuged (described in Materials and Methods). The vesicle-free supernatant was collected after the final ultracentrifugation. The mock control and the vesicle-free supernatant were stored under the same conditions as the isolated EcO83-OMVs until further use.

**Testing EcO83-OMVs, mock control and EcO83-OMVs-depleted supernatant for bacterial contamination**

50 µl EcO83-OMVs (diluted 1:5 with NaCl), a mock control (diluted 1:5 with NaCl) and the supernatant depleted in EcO83-OMVs (undiluted) were applied to LB agar plates (LB medium containing 1.5 % agarose; both Carl Roth) and incubated at 37 °C for 96 h. The plates were examined every 24 h for visible bacterial colonies.

**Culture of lung cells with mock control, EcO83-OMVs-depleted supernatant, Polymyxin B- and heat-treated EcO83-OMVs**

Lung cells were isolated as described in Materials and Methods and seeded at a concentration of 5 x 10^6^ cells/ml. EcO83-OMVs (1 ng/ml, 10 ng/ml, 100 ng/ml), medium, LPS (1 µg/ml), *E. coli* O83 (10^8^ CFU/ml), the mock control and EcO83-OMVs-depleted supernatant were added to the cultures. The mock control and EcO83-OMVs-depleted supernatant were used in the amount equivalent to the amount of EcO83-OMVs used for the culture at 100 ng/ml. To block TLR4 signalling by LPS, EcO83-OMVs were incubated with 20 µg/ml Polymyxin B (Sigma-Aldrich) at 37 °C for 30 minutes, as previously described [1]. As a mock control, EcO83-OMVs were incubated 30 min at 37 °C without Polymyxin B. Lung cells were stimulated with the Polymyxin B-treated EcO83-OMVs (1 ng/ml, 10 ng/ml, 100 ng/ml), medium, LPS (1 µg/ml) and *E. coli* O83 (10^8^ CFU/ml). In parallel, EcO83-OMVs were incubated at 95 °C for 15 min and added to lung cells as described above. The cells were incubated for 48 h at 37 °C and 5 % CO_2_. The cell culture supernatants were collected and stored at -20 °C until further use. The levels of IL-6 and TNF-α in the cell cultures were measured by ELISA.

**Cultivation and stimulation of the macrophage-like cell line**

The macrophage-like cell line RAW264.7 (a gift from Prof. Sylvia Knapp) was cultured in DMEM medium with 10 % FCS at 37 °C/5 % CO_2_. In general, the growth medium was changed every 2-3 days and the cultures were divided when they reached ~80 % confluence. For stimulation, cells were plated out in 96-well plates at a concentration of 2 x 10^5^ cells /ml and allowed to adhere for 16 h. Cells were then stimulated for 6 h at 37 °C/5 % CO_2_ with medium, LPS (1 µg/ml) and EcO83-OMVs (10 ng/ml and 100 ng/ml)_._ The supernatant was discarded and the cells were scraped from the surface with a pipette tip, collected and transferred to 100 µl RNAlater solution (Thermo Fisher).

**RNA isolation**

RNA was isolated from stimulated RAW264.7 cells using the innuPREP RNA Mini Kit 2.0 (Analytik Jena) according to the manufacturer's protocol for eukaryotic cells. Subsequently, the purity and concentration of the extracted RNA were quantified using a NanoDrop 2000 spectrophotometer (PreqLab).

###

**Reverse transcription and quantitative real-time PCR (qRT-PCR)**

To initiate cDNA synthesis, 1 µg of total RNA was subjected to reverse transcription using the iScript cDNA Synthesis Kit (Bio-Rad). The mixture was incubated in a thermal cycler (Bio-Rad T100^TM^). Real-time qPCR was performed with the SsoAdvanced Universal SYBR® Green Supermix (Bio Rad) using the CFX Duet (Bio Rad). The thermal cycles consisted of denaturation 98 °C/3 min, 44 amplification cycles at 95 °C/10 s and 60 °C/60 s, followed by melting at 60-95 °C/5 s. The primer sequences for the tested genes (Eurofins Genomics) are listed below:

***IL-1β:*** 5’-GAT CCA CAC TCT CCA GCT GCA-3’ (forward); 5’-CAA CCA ACA AGT GAT ATT CTC CAT-3’ (reverse)

***IL-6:***

5’-CAC TTC ACA AGT CGG AGG CT-3’ (forward); 5’-CTG CAA GTG CAT CAT CGT TGT-3’ (reverse)

***TNF-α:***

5’-TGC CTA TGT CTC AGC CTC TTC-3’ (forward); 5’-TTG TGA GTG TGA GGG TCT GG-3’ (reverse)

***GAPDH:***

5’-AAC TTT GGC ATT GTG GAA GG-3’ (forward); 5’-ACA CAT TGG GGG TAG GAA CA-3’ (reverse)

Samples were analysed in duplicates. Fold expression change was calculated by the comparative 2-ΔΔCt method using CFX Manager software version 2.3 (Bio Rad), using glyceraldehyde-3-phosphate dehydrogenase (GAPDH) as the reference gene for normalisation.

**SUPPLEMENTARY RESULTS**

**Table S2: Size distribution and concentration of vesicles and amount of protein, and endotoxin corresponding to material collected from a two litre culture.** Average ± SD is shown from three independent measurements.

| **Measured Parameter** | **Method** | **Values** |
| --- | --- | --- |
| Particle size | Dynamic Light Scattering | 112.9 ± 1.99 nm |
| Particle concentration | Dynamic Light Scattering | 1.4 x 10^13^ particles/ml (7 x 10^12^ particles) |
| Protein content | Bradford | 1.84 ± 0,12 mg/ml (~0.92 mg) |
| LPS content | rFC assay | 5 mg LPS/mg protein (~4.6 mg) |

**Table S3: Protein content in EcO83-OMVs measured with Bioanalyzer**. Marked in grey are dominant visible bands.

| **Size [kDa]** | **Relative Concentration [ng/µl]** | **% of Total** | **Comments** |
| --- | --- | --- | --- |
| 0 | 0 | 0 |  |
| 0,3 | 0 | 0 |  |
| 4,5 | 0 | 0 | Lower Marker |
| 10 | 0 | 0 | System Peak |
| 12,2 | 0 | 0 | System Peak |
| 19 | 172,6 | 19,5 |  |
| 27 | 94,3 | 10,7 |  |
| 34,3 | 207,4 | 23,5 |  |
| 49 | 77,6 | 8,8 |  |
| 64,6 | 242,9 | 27,5 |  |
| 77,5 | 12,8 | 1,4 |  |
| 115,6 | 29,2 | 3,3 |  |
| 178,3 | 0,9 | 0,1 |  |
| 193,7 | 5,9 | 0,7 |  |
| 209,1 | 16,4 | 1,9 |  |
| 224,6 | 24,2 | 2,6 |  |
| 240 | 60 | 0 | Upper Marker |
| 394,3 | 0 | 0 |  |

**Table S4: List of proteins that were found to be upregulated in EcO83-OMVs compared to *E. coli* O83 lysate and met the false discovery rate (FDR) criteria.** (P-value<0.05). n.d. = not determined.

| **UniProt accession** | **Entry name** | **Name** | **Localization** | **Log_2_ (Fold change)** | **- Log_10_ (p-value)** |
| --- | --- | --- | --- | --- | --- |
| P33235 | FLGK_ECOLI | Flagellar hook-associated protein 1 | cell envelope | 33,90 | 7,85 |
| P29744 | FLGL_ECOLI | Flagellar hook-associated protein 3 | cell envelope | 32,46 | 9,08 |
| P0A927 | TSX_ECOLI | Nucleoside-specific channel-forming protein Tsx | outer membrane | 30,88 | 6,39 |
| P13036 | FECA_ECOLI | Fe(3+) dicitrate transport protein FecA | outer membrane | 30,76 | 9,52 |
| P75937 | FLGE_ECOLI | Flagellar hook protein FlgE | cell envelope | 29,18 | 8,40 |
| P77717 | YBAY_ECOLI | Uncharacterized lipoprotein YbaY | cell membrane | 28,67 | 7,42 |
| P37665 | YIAD_ECOLI | Probable lipoprotein YiaD | inner, outer membrane | 28,31 | 8,27 |
| P10100 | RLPA_ECOLI | Endolytic peptidoglycan transglycosylase RlpA | cell membrane | 28,16 | 8,81 |
| P0A921 | PA1_ECOLI | Phospholipase A1 | outer membrane | 27,92 | 9,36 |
| P0ABX5 | FLGG_ECOLI | Flagellar basal body rod protein FlgG | cell envelope | 27,78 | 7,02 |
| P0A935 | MLTA_ECOLI | Membrane-bound lytic murein transglycosylase A | outer membrane | 27,75 | 7,80 |
| P02943 | LAMB_ECOLI | Maltoporin | outer membrane | 27,71 | 8,41 |
| P10384 | FADL_ECOLI | Long-chain fatty acid transport protein | outer membrane | 27,70 | 8,12 |
| P45464 | LPOA_ECOLI | Penicillin-binding protein activator LpoA | outer membrane | 27,60 | 9,85 |
| P76115 | YNCD_ECOLI | Probable TonB-dependent receptor YncD | outer membrane | 27,43 | 9,27 |
| P77774 | BAMB_ECOLI | Outer membrane protein assembly factor BamB | outer membrane | 27,23 | 7,44 |
| P0ADA7 | OSMB_ECOLI | Osmotically-inducible lipoprotein B | cell membrane | 27,13 | 7,86 |
| P0ABW9 | FLGB_ECOLI | Flagellar basal body rod protein FlgB | cell envelope | 26,93 | 7,85 |
| P0ABX2 | FLGC_ECOLI | Flagellar basal body rod protein FlgC | cell envelope | 26,79 | 8,05 |
| P61320 | LOLB_ECOLI | Outer-membrane lipoprotein LolB | outer membrane | 26,66 | 8,75 |
| P60785 | LEPA_ECOLI | Elongation factor 4 | inner membrane | 26,62 | 9,64 |
| P0C960 | EMTA_ECOLI | Endo-type membrane-bound lytic murein transglycosylase A | outer membrane | 26,53 | 6,55 |
| P76206 | YDIY_ECOLI | Uncharacterized protein YdiY | n.d. | 26,39 | 8,68 |
| P0AC02 | BAMD_ECOLI | Outer membrane protein assembly factor BamD | outer membrane | 25,89 | 12,55 |
| P52614 | FLIK_ECOLI | Flagellar hook-length control protein | cell envelope | 25,87 | 9,21 |
| Q46798 | YGER_ECOLI | Uncharacterized lipoprotein YgeR | inner membrane | 25,80 | 6,75 |
| P08189 | FIMF_ECOLI | Protein FimF | cell envelope/secreted | 25,80 | 6,61 |
| P0ADC1 | LPTE_ECOLI | LPS-assembly lipoprotein LptE | outer membrane | 25,78 | 8,34 |
| P08190 | FIMG_ECOLI | Protein FimG | cell envelope | 25,77 | 6,38 |
| P75780 | FIU_ECOLI | Catecholate siderophore receptor Fiu | outer membrane | 25,59 | 9,01 |
| P0AA16 | OMPR_ECOLI | DNA-binding dual transcriptional regulator OmpR | cytoplasm | 25,56 | 9,32 |
| P65292 | YGDI_ECOLI | Uncharacterized lipoprotein YgdI | cell membrane | 25,50 | 7,63 |
| P76506 | MLAA_ECOLI | Intermembrane phospholipid transport system lipoprotein MlaA | outer membrane | 25,20 | 8,10 |
| P11454 | ENTF_ECOLI | Enterobactin synthase component F | cytoplasm | 25,15 | 8,40 |
| P0ACR9 | MPRA_ECOLI | Transcriptional repressor MprA | cytosol | 24,94 | 7,75 |
| P77294 | YDER_ECOLI | Uncharacterized fimbrial-like protein YdeR | cell envelope | 24,93 | 8,80 |
| P0A9E5 | FNR_ECOLI | Fumarate and nitrate reduction regulatory protein | cytoplasm | 24,92 | 8,14 |
| P39264 | FIMI_ECOLI | Fimbrin-like protein FimI | cell envelope | 24,92 | 7,54 |
| Q7DFV3 | YMGG_ECOLI | UPF0757 protein YmgG | n.d. | 24,79 | 8,84 |
| P0A853 | TNAA_ECOLI | Tryptophanase | cytoplasm | 24,66 | 7,91 |
| P0A6R3 | FIS_ECOLI | DNA-binding protein Fis | cytoplasm | 24,56 | 7,10 |
| P77562 | YAIW_ECOLI | Uncharacterized protein YaiW | outer membrane, cytosol | 24,53 | 6,85 |
| P0AAB6 | GALF_ECOLI | UTP--glucose-1-phosphate uridylyltransferase | cytosol | 24,49 | 8,22 |
| P0ACJ0 | LRP_ECOLI | Leucine-responsive regulatory protein | cytosol | 24,46 | 9,29 |
| P41052 | MLTB_ECOLI | Membrane-bound lytic murein transglycosylase B | outer membrane | 24,45 | 9,76 |
| P0ADG4 | SUHB_ECOLI | Nus factor SuhB | cytoplasm | 24,33 | 9,41 |
| P0ACP7 | PURR_ECOLI | HTH-type transcriptional repressor PurR | cytosol | 24,32 | 6,65 |
| P0A9G6 | ACEA_ECOLI | Isocitrate lyase | cytosol | 24,32 | 7,70 |
| P75938 | FLGF_ECOLI | Flagellar basal-body rod protein FlgF | cell envelope | 24,28 | 8,64 |
| P46130 | YBHC_ECOLI | Putative acyl-CoA thioester hydrolase YbhC | outer membrane | 24,15 | 6,90 |
| P0A6E9 | BIOD2_ECOLI | ATP-dependent dethiobiotin synthetase BioD 2 | cytoplasm | 24,13 | 8,64 |
| P0A7D1 | PTH_ECOLI | Peptidyl-tRNA hydrolase | cytoplasm | 24,05 | 7,29 |
| P0A887 | UBIE_ECOLI | Ubiquinone/menaquinone biosynthesis C-methyltransferase UbiE | cytoplasm | 24,03 | 6,40 |
| P0A6S0 | FLGH_ECOLI | Flagellar L-ring protein | cell envelope | 23,97 | 8,49 |
| P0C058 | IBPB_ECOLI | Small heat shock protein IbpB | cytoplasm | 23,86 | 6,88 |
| P76537 | YFEY_ECOLI | Uncharacterized protein YfeY | n.d. | 23,79 | 7,03 |
| P0AA91 | YEAY_ECOLI | Uncharacterized lipoprotein YeaY | cell membrane | 23,68 | 7,56 |
| P39180 | AG43_ECOLI | Antigen 43 | periplasm, secreted | 23,60 | 5,72 |
| P69924 | RIR2_ECOLI | Ribonucleoside-diphosphate reductase 1 subunit beta | cytoplasm, cytosol | 23,59 | 10,60 |
| P77804 | YDGA_ECOLI | Protein YdgA | inner membrane | 23,45 | 7,59 |
| P0ADN6 | YIFL_ECOLI | Uncharacterized lipoprotein YifL | cell membrane | 23,39 | 6,10 |
| P0AEE5 | DGAL_ECOLI | D-galactose/methyl-galactoside binding periplasmic protein MglB | periplasm | 23,38 | 7,21 |
| P63883 | AMIC_ECOLI | N-acetylmuramoyl-L-alanine amidase AmiC | periplasm | 22,96 | 9,05 |
| P0A8I3 | YAAA_ECOLI | Peroxide stress resistance protein YaaA | cytosol | 22,96 | 6,15 |
| P0CG19 | RNPH_ECOLI | Truncated inactive ribonuclease PH | cytosol | 22,93 | 6,82 |
| P0C054 | IBPA_ECOLI | Small heat shock protein IbpA | cytoplasm | 22,73 | 9,79 |
| P0ACP1 | CRA_ECOLI | Catabolite repressor/activator | n.d. | 22,68 | 8,08 |
| P0A734 | MINE_ECOLI | Cell division topological specificity factor | cytosol, plasma membrane | 22,66 | 7,62 |
| P21362 | YCIF_ECOLI | Protein YciF | n.d. | 22,56 | 9,45 |
| P30958 | MFD_ECOLI | Transcription-repair-coupling factor | cytoplasm | 22,51 | 9,05 |
| P46837 | YHGF_ECOLI | Protein YhgF | cytosol | 22,36 | 8,53 |
| P64624 | YHEO_ECOLI | Uncharacterized protein YheO | cytosol | 22,31 | 6,70 |
| P18196 | MINC_ECOLI | Septum site-determining protein MinC | cell pole, cytosol | 21,98 | 7,69 |
| P09551 | ARGT_ECOLI | Lysine/arginine/ornithine-binding periplasmic protein | periplasm | 21,92 | 4,84 |
| P27550 | ACSA_ECOLI | Acetyl-coenzyme A synthetase | cytosol | 21,77 | 8,40 |
| P37650 | BCSC_ECOLI | Cellulose synthase operon protein C | outer membrane | 21,56 | 5,22 |
| P66948 | BEPA_ECOLI | Beta-barrel assembly-enhancing protease | periplasm | 21,15 | 6,81 |
| P31554 | LPTD_ECOLI | LPS-assembly protein LptD | outer membrane | 19,68 | 1,31 |
| P09169 | OMPT_ECOLI | Protease 7 | outer membrane | 10,40 | 3,68 |
| P0A940 | BAMA_ECOLI | Outer membrane protein assembly factor BamA | outer membrane | 7,86 | 4,55 |
| P0A908 | MIPA_ECOLI | MltA-interacting protein | outer membrane | 7,69 | 3,90 |
| P06971 | FHUA_ECOLI | Ferrichrome outer membrane transporter/phage receptor | outer membrane | 7,50 | 3,96 |
| P77330 | BORD_ECOLI | Prophage lipoprotein Bor homolog | cell membrane | 6,98 | 5,10 |
| P04949 | FLIC_ECOLI | Flagellin | cell envelope | 6,91 | 4,92 |
| P64596 | YRAP_ECOLI | Outer membrane lipoprotein DolP | outer membrane | 6,79 | 5,43 |
| P0A912 | PAL_ECOLI | Peptidoglycan-associated lipoprotein | outer mmebrane | 6,46 | 4,55 |
| P0A910 | OMPA_ECOLI | Outer membrane protein A | outer membrane | 6,06 | 5,02 |
| P0A917 | OMPX_ECOLI | Outer membrane protein X | outer membrane | 5,83 | 5,74 |
| P21420 | NMPC_ECOLI | Putative outer membrane porin protein NmpC | outer membrane | 5,60 | 4,07 |
| P0A707 | IF3_ECOLI | Translation initiation factor IF-3 | cytoplasm | 5,58 | 5,40 |
| P69776 | LPP_ECOLI | Major outer membrane lipoprotein Lpp | outer membrane, secreted, cell wall | 4,96 | 4,22 |
| P0ADB1 | OSME_ECOLI | Osmotically-inducible putative lipoprotein OsmE | inner membrane | 4,92 | 3,91 |
| P06996 | OMPC_ECOLI | Outer membrane porin C | outer membrane | 4,82 | 4,48 |
| P0ADA5 | YAJG_ECOLI | Uncharacterized lipoprotein YajG | cell membrane | 4,73 | 3,34 |
| P0A915 | OMPW_ECOLI | Outer membrane protein W | outer membrane | 4,46 | 3,79 |
| P76513 | YFDQ_ECOLI | Uncharacterized protein YfdQ | n.d. | 4,29 | 5,58 |
| P0A905 | SLYB_ECOLI | Outer membrane lipoprotein SlyB | outer membrane | 4,18 | 4,80 |
| P0A903 | BAMC_ECOLI | Outer membrane protein assembly factor BamC | outer membrane | 4,07 | 3,61 |
| P0ACJ8 | CRP_ECOLI | cAMP-activated global transcriptional regulator CRP | cytosol | 3,79 | 4,93 |
| P0A7G6 | RECA_ECOLI | Protein RecA | cytoplasm | 3,78 | 2,95 |
| P0ADA3 | NLPD_ECOLI | Murein hydrolase activator NlpD | inner membrane | 3,39 | 3,83 |
| P0A7M2 | RL28_ECOLI | 50S ribosomal protein L28 | cytoplasm, cytosol | 3,27 | 3,55 |
| P0AAX8 | YBIS_ECOLI | Probable L,D-transpeptidase YbiS | periplasm | 3,20 | 3,97 |
| P68688 | GLRX1_ECOLI | Glutaredoxin 1 | n.d. | 3,14 | 2,57 |
| P0A8F0 | UPP_ECOLI | Uracil phosphoribosyltransferase | cytosol, cytoplasm | 3,12 | 4,60 |
| P60716 | LIPA_ECOLI | Lipoyl synthase | cytoplasm | 3,08 | 3,75 |
| P0AB89 | PUR8_ECOLI | Adenylosuccinate lyase | cytosol | 3,03 | 2,24 |
| P37194 | SLP_ECOLI | Outer membrane protein Slp | outer membrane | 2,67 | 3,97 |
| P0A7G2 | RBFA_ECOLI | 30S ribosome-binding factor | cytoplasm | 2,24 | 3,54 |
| P0A9Q1 | ARCA_ECOLI | Aerobic respiration control protein ArcA | cytoplasm | 2,18 | 3,32 |
| P0A7V3 | RS3_ECOLI | 30S ribosomal protein S3 | cytoplasm, cytosol | 2,16 | 3,71 |

**SUPPLEMENTARY FIGURES**

**(A)**

Figure S1_Schmid *et al.*


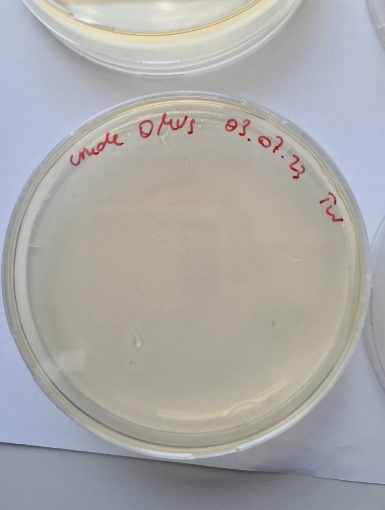

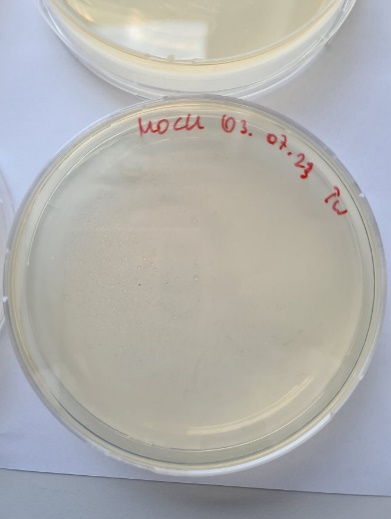

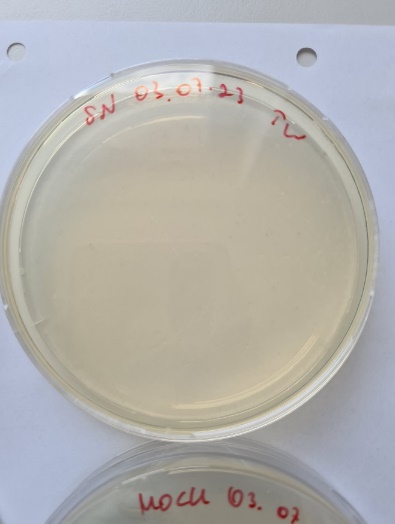


EcO83-OMVs

Mock

Supernatant

Figure S2_Schmid *et al.*

Figure S3_Schmid *et al.*

Figure S4_Schmid *et al.*

Figure S5_Schmid *et al.*

**

Figure S6_Schmid *et al.*

Figure S7_Schmid *et al.*


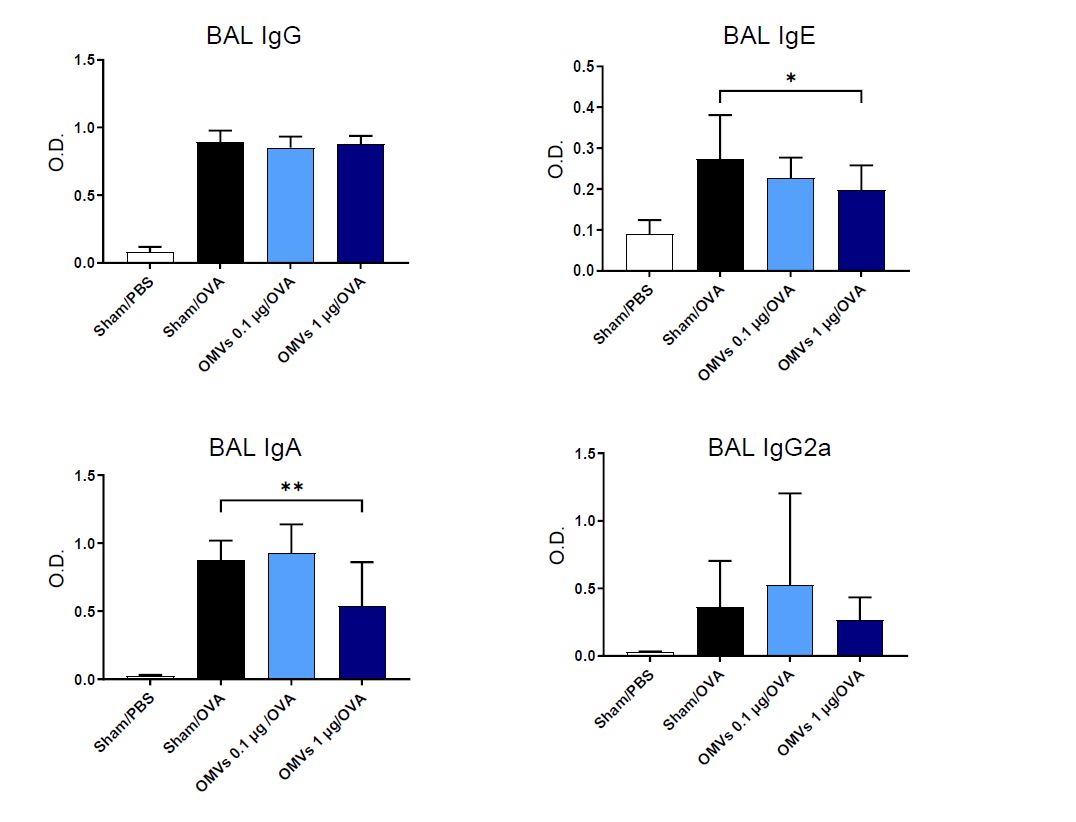


Figure S8_Schmid *et al.*

Serum IgE

Serum IgA

**(A)**

Spleen IFN-γ

Spleen IL-10

**(B)**

Spleen IL-4

Spleen IL-5

Serum IgG2a

Serum IgG1

**SUPPLEMENTARY FIGURE LEGENDS**

**Figure S1: Examination of EcO83-OMVs, EcO83-OMVs-depleted supernatant and mock control for bacterial contamination. The isolated** EcO83-OMVs, EcO83-OMVs-depleted supernatant and mock control were examined for the presence of live bacteria. Samples were placed on LB agar plates and incubated at 37 °C for 96 h. The plates were examined for visible bacterial colonies.

**Figure S2: Unprocessed image of Bioanalyzer protein analysis of EcO83-OMVs and *E. coli* O83 lysate (modified image in Fig. 1E).** Samples 1 and 2: EcO83-OMVs in duplicates. Samples 9 and 10: *E. coli* O83 lysate in duplicates. Samples 3-8: Material not relevant to this publication.

**Figure S3: Activation of the NF-κB pathway by EcO83-OMVs.** The macrophage-like mouse cell line RAW264.7 was stimulated with medium, LPS (1 µg/ml), *E. coli* O83 (10^7^ CFU/ml) and EcO83-OMVs (10 ng/ml and 100 ng/ml) at 37 °C and 5 % CO_2_ for 6 h. The expression of IL-6, TNF-α and IL-1β mRNA was measured with RT-PCR and is presented as the fold change to the housekeeping gene GAPDH. OMVs = EcO83-OMVs

**Figure S4:** **Immunostimulatory potential of EcO83-OMVs compared to EcO83-OMVs-depleted supernatant and mock control.** Cells isolated from the lungs of naive mice (n = 5) were treated with medium, LPS (1 µg/ml), *E. coli* O83 (10^8^ CFU/ml), EcO83-OMVs (1 ng/ml, 10 ng/ml, 100 ng/ml), EcO83-OMVs-depleted supernatant and mock control. The supernatant and mock control were added in the amount equal to the volume of vesicles used in cultures with 100 ng/ml EcO83-OMVs and incubated at 37 °C and 5 % CO_2_ for 48 h. IL-6 and TNF-α were measured in the cell culture supernatant by ELISA. The mean ± SD is shown. Data were analysed using a One-Way ANOVA followed by a post-hoc Tukey’s multiple comparison test. **p<0.05; ****p<0.001. OMVs = EcO83-OMVs.

**Figure S5: Immunostimulatory potential of EcO83-OMVs compared to Polymyxin B-treated EcO83-OMVs.** Cells isolated from the lungs of naive mice (n = 5) were treated with medium, LPS (1 µg/ml), *E. coli* O83 (10^8^ CFU/ml), EcO83-OMVs treated with Polymyxin B (1 ng/ml, 10 ng/ml, 100 ng/ml), EcO83-OMVs treated with Polymyxin B mock (1 ng/ml, 10 ng/ml, 100 ng/ml) and stimulated with Polymyxin B alone at 37 °C/ 5% CO_2_ for 48 h. IL-6 and TNF-α were measured in the supernatants by ELISA. The mean ± SD is shown. Data were analysed using a One-Way ANOVA followed by a post-hoc Tukey’s multiple comparison test. ***p < 0.01; ****p < 0.001. OMVs = EcO83-OMVs ; PmB = Polymyxin B

**Fig. S6: Immunostimulatory potential of EcO83-OMVs compared to heat-treated EcO83-OMVs.** Cells isolated from the lungs of naive mice (n = 5) were treated with medium, LPS (1 µg/ml), *E. coli* O83 (10^8^ CFU/ml), EcO83-OMVs (1 ng/ml, 10 ng/ml, 100 ng/ml) and heat-treated EcO83-OMVs (1 ng/ml, 10 ng/ml, 100 ng/ml) at 37 °C/ 5 % CO_2_ for 48 h. IL-6 and TNF-α were measured in the supernatants by ELISA. The mean ± SD is shown. Data were analysed using a One-Way ANOVA followed by a post-hoc Tukey’s multiple comparison test. ****p<0.001. OMVs = EcO83-OMVs

**Figure S7: Local allergen-specific responses: Levels of OVA-specific antibodies in BAL.** BAL was collected on sacrifice day by flushing the lungs with PBS and OVA-specific antibodies were measured by ELISA. Data were analysed using a One-Way ANOVA followed by post-hoc Tukey’s multiple comparison test. *p<0.5; **p<0.05. n = 5/group. Data are representative of three independent experiments. Mean ± SD is shown. Significant differences between Sham/OVA and EcO83-OMVs treatment groups (OMVs 0.1 µg/OVA or OMVs 1 µg/OVA) are indicated. BAL = bronchoalveolar lavage; OVA = ovalbumin; OMVs = EcO83-OMVs.

**Figure S8:** **Systemic allergen-specific responses:** **Levels of OVA-specific antibodies in serum and cytokines produced by OVA-stimulated splenocytes *ex vivo.*** **(A)** Blood was sampled on days -1, 20 and 26 (Experimental setup see Figure 5 A) and centrifuged to obtain serum. OVA-specific antibodies in serum were measured by ELISA. **(B)** Single cell suspensions of excised spleens of Sham or EcO83-OMVs-treated allergic mice were stimulated with OVA for 72 h and cytokine levels were measured by ELISA. Data was analysed using a One-Way ANOVA followed by post-hoc Tukey’s multiple comparison test. *****p<0.5; **p<0.05; ***p<0.001. **(A)** Data are representative of three independent experiments for samples collected on day -1 and a pool of two experiments for samples collected on days 20 and 26; n = 5-10/group. **(B)** Data are representative of three independent experiments, n = 5/group. Mean ± SD is shown. Significant differences between Sham/OVA and EcO83-OMVs treatment groups (OMVs 0.1 µg/OVA or OMVs 1 µg/OVA) are indicated. OVA = ovalbumin; OMVs = EcO83-OMVs.

**SUPPLEMENTARY REFERENCES**

1. Korb, E. *et al.* Reduction of Allergic Lung Disease by Mucosal Application of *Toxoplasma gondii*-Derived Molecules: Possible Role of Carbohydrates. *Front Immunol* **11**, (2021).
